# A geometric analysis of the SIRS epidemiological model on a homogeneous network

**DOI:** 10.1007/s00285-021-01664-5

**Published:** 2021-09-22

**Authors:** Hildeberto Jardón-Kojakhmetov, Christian Kuehn, Andrea Pugliese, Mattia Sensi

**Affiliations:** 1 Faculty of Science and Engineering, University of Groningen, Groningen, The Netherlands; 2 Department of Mathematics, Technical University of Munich, Munich, Germany; 3 Università degli Studi di Trento, Trento, Italy

**Keywords:** Fast–slow system, Epidemic model, Non-standard form, Epidemics on networks, Bifurcation analysis, 34C23, 34C60, 34E13, 34E15, 37N25, 92D30

## Abstract

We study a fast–slow version of an SIRS epidemiological model on homogeneous graphs, obtained through the application of the moment closure method. We use GSPT to study the model, taking into account that the infection period is much shorter than the average duration of immunity. We show that the dynamics occurs through a sequence of fast and slow flows, that can be described through 2-dimensional maps that, under some assumptions, can be approximated as 1-dimensional maps. Using this method, together with numerical bifurcation tools, we show that the model can give rise to periodic solutions, differently from the corresponding model based on homogeneous mixing.

## Introduction

Mathematical epidemics modelling is, now more than ever, an important and urgent field to explore. A deep understanding of how diseases evolve and spread can give, and has given, us strategies to contain, treat and even prevent them. Over the years, mathematical modellers have made a variety of different assumptions, in order to obtain a tractable trade-off between simplicity, which allows for more in-depth analysis, and realism, which allows to make more precise predictions.

In particular, compartment models build on the core idea that the population can, at any time, be portioned into compartments characterized by a specific state with respect to the ongoing epidemic. The first of such models divides the population into Susceptible, Infected and Recovered individuals, from which the SIR acronym is used. A Susceptible can become Infected ($S \rightarrow I$) by making contact with an already infected individual, and can then either Recover ($I \rightarrow R$) or die, if we assume the disease to be characterized by permanent immunity after a first infection. If we do not make such an assumption, and allow recovered individuals to become susceptible again ($R \rightarrow S$), we obtain a so called SIRS model. Many more models, with different compartments, have been proposed and analysed in the past, see e.g. Dafilis et al. (2012) and Hethcote (2000).

Classical compartmental models are based on the homogeneous mixing assumption, i.e. the assumption that any individual in a population may have contacts with any other. Such an assumption, however, is quite unrealistic for many situations in which the observed population is large, and possibly divided in classes, families or generally sub-populations. One possible extension is to subdivide the population into groups, assuming homogeneous mixing within each group, but representing inter-group interactions through a contact matrix (Mossong et al. 2008). Another possible approach is to take into account the network structure of contacts. Often, epidemic dynamics on a network is analysed only through simulations (López-García 2016; Smilkov et al. 2014; Zhang et al. 2013; Castellano and Pastor-Satorras 2010; Volz 2008; Ganesh et al. 2005). The method of pair approximations, introduced in epidemiology by Satō et al. (1994) and Keeling et al. (1997), allows to build a system of differential equations that retains some aspects of the network structure. The ideas and some applications of the methods are presented in detail in the monograph by Kiss et al. (2017). However, not much analytical progress has been made in the study of the resulting systems, possibly because they are generally rather complex.

This paper aims at introducing methods from Geometric Singular Perturbation Theory (GSPT) to analyse these systems, building on the ideas introduced in Jardón-Kojakhmetov et al. (2021). The difference in time-scales between epidemic spread and demographic turnover, which can be observed in many diseases, is the motivation for the use of techniques from GSPT. We refer to Jardón-Kojakhmetov et al. (2021) for a brief introduction of the techniques we use, or to the references therein, and in particular to Jones (1995) and Kuehn (2015), for a more detailed explanation. In particular, we will exploit the *entry–exit function* (De Maesschalck 2008; De Maesschalck and Schecter 2016) to analyse the behaviour of the system on its critical manifold, which is characterized by a change in stability over a hyperplane.

In this work, we assume homogeneity of the network, in order to obtain analytical results, before validating them numerically. Even with such an assumption, the additional complexity brought by the network structure must be treated properly. In fact, in order to completely describe the evolution of a network in time, one needs to have an equation for each possible state of its nodes, one for each possible state of its edges (along which the epidemic spreads), one for each possible state of triples, i.e. three nodes connected by two edges, and so on. This procedure, however, would generate very large system of ODEs, which would once again be hardly treatable with analytical tools. In order to overcome this difficulty, one can apply the so-called *moment closure* (Kiss et al. 2017; Kuehn 2016), i.e. approximation formulas which allow us to truncate the dimension of the objects we want to analyse. If we truncate at the node level, we lose the network structure, and we recover a homogeneously mixing system. Instead, we truncate at the edge level, using the pair approximation discussed above, and analyse the system which derives from this choice.

To our knowledge, there are relatively few articles in which GSPT has been applied rigorously to epidemics models (Rocha et al. 2016; Jardón-Kojakhmetov et al. 2021; Heesterbeek and Metz 1993; Zhang et al. 2009; Brauer 2019; Wang et al. 2014). However, for most infectious diseases, the presence of different time scales is natural. Moreover, though a SIR model on networks has been studied with moment closure already (Bidari et al. 2016; Kiss et al. 2017), the SIRS extension has not. Likewise, a thorough bifurcation analysis on compartment models such as the one we analyse in this paper is not present in the literature. The additional feature of the network structure, even in its most simplified version, i.e. homogeneous network, unravels new dynamics for the SIRS system we study. Indeed, we show that there exists a set in the parameter space which allows the system to exhibit a stable limit cycle, a situation that does not arise in the classical compartmental SIRS model. To complement the bifurcation analysis, we extend the geometrical argument from Jardón-Kojakhmetov et al. (2021) to the higher dimensional system we study, providing additional justification for the existence of stable limit cycles. It is worth noticing that the model we study is not globally in fast–slow standard form; as in Jardón-Kojakhmetov et al. (2021); Kuehn and Szmolyan (2015); Kosiuk and Szmolyan (2016), the fast–slow dynamics are only evident in specific regions of the phase space, in which a local change of coordinates brings the system to a standard two time scales form. In particular, we refer to the very recent monograph (Wechselberger 2020), in which the properties of singularly perturbed systems in non-standard form are thoroughly analysed.

This article builds on the analysis on a homogeneous mixing SIRS model we carried out in Jardón-Kojakhmetov et al. (2021), generalizing it to a more complex setting. The main novelty we introduce is the network structure, which increases both the dimensionality and the complexity of the ODE system we study. This additional feature, which increases the realism of the model, allows us to unveil additional dynamics, namely stable limit cycles. Lastly, a further challenge is posed by the fact that the rescaled system close to the critical manifold is characterized by multiple fast variables, as compared to a single fast variable which characterized all the systems studied in Jardón-Kojakhmetov et al. (2021).

The paper is structured as follows: in Sect. 2, we recall the derivation of the model, and introduce the moment closure technique. In Sect. 3, we obtain analytical results on the model, in particular on the fast and slow limit systems and on the application of the entry–exit function. In Sect. 4, we perform a bifurcation analysis and numerical exploration of the model. Finally, in Sect. 5, we conclude with a summary of the results, and with possible research outlooks.

## Formulation of the SIRS model on a network

In this section we describe and propose an SIRS model for epidemics on graphs, building on the model proposed in Kiss et al. (2017, Sec. 4.2.2). We are interested in the graph generalization of the model studied in Jardón-Kojakhmetov et al. (2021), in order to drop the homogeneous-mixing hypothesis, under which we assumed that each individual in the population could have contacts with any other. We then assume loss of immunity to be slower, compared to the other rates (this is the case e.g. for pertussis (Dafilis et al. 2012; Lavine et al. 2011), and it could potentially be true for the recent SARS-CoV-2 (Kissler et al. 2020; Randolph and Barreiro 2020)); this assumption brings the model to a non-standard singularly perturbed system of ODEs, which we study with techniques from GSPT.

### The model

The construction of the model is essentially what is presented in detail in Kiss et al. (2017, Ch. 4), extended to the SIRS case. For ease of reading, we briefly repeat the whole method.

We consider a network of *N* nodes, with *N* large, representing the individuals of a population, and we assume this network to be homogeneous, meaning that each node has fixed degree $n \in {\mathbb {N}}_{\ge 2}$, representing the number of direct neighbours each individual has. We assume the network to be undirected and completely connected, meaning that, given any two nodes in the network, there is a finite sequence of edges (or an *undirected path*) which starts in the first and ends in the second. Each node can be in one of either three states, namely *S* (susceptible), *I* (infected) or *R* (recovered). We will indicate the number of each state at time *t* with $[\cdot ](t)$; we stress the distinction between the notation *X*, indicating a state, and [*X*], indicating the number of individuals in the state *X*. We indicate the number of edges connecting a node in state *X* to one in state *Y* at time *t* with [*XY*](*t*) for all $t\ge 0$. We distinguish between an edge *XY*, counted starting from a node in state *X*, and the same edge counted starting from the other node in state *Y*, for a reason of conserved quantities, namely (7a), (7b) and (7c) to be defined below. For example, we count the number of edges *SI* by “visiting” each node in state *S*, and counting all its neighbours in state *I*, then summing over all the nodes in state *S*; this implies that, at all times, by definition, $[SI]=[IS]$. The edges connecting a node with another in the same state, such as *SS*, hence, will always be counted twice.

Infection can only spread if a node in state *S* is connected to a node in state *I* through an edge *SI*; we denote the infection rate with $\beta \ge 0$. Nodes in state *I* recover, independently from their neighbours, at a rate $\gamma >0$; and nodes in state *R* lose their immunity, again independently from their neighbours, at a slow rate $\epsilon$, with $0<\epsilon \ll \beta ,\gamma$. Based upon these modelling assumptions, it is then straightforward to prove using the master equation of the epidemic model, that one obtains the following system of ODEs:

1 $$\begin{aligned} \begin{aligned} {[S]}'&={}-\beta [SI]+ \epsilon [R],\\ {[I]}'&={}\beta [SI]- \gamma [I],\\ {[R]}'&={}\gamma [I]- \epsilon [R].\\ \end{aligned} \end{aligned}$$

From our assumptions, the sum of $[S]+[I]+[R]\equiv N$ is conserved at all times; we normalize by dividing both nodes and edges by *N*, and we do not rename the new variables, which now indicate the density of nodes, and a rescaled fraction of edges, in each state. Now $[S]+[I]+[R]\equiv 1$, so we can reduce the dimension of system (1) by removing [*R*], obtaining the system

2 $$\begin{aligned} \begin{aligned} {[S]}'&={}-\beta [SI]+ \epsilon (1-[S]-[I]),\\ {[I]}'&={}\beta [SI]- \gamma [I].\\ \end{aligned} \end{aligned}$$

**Fig. 1:**
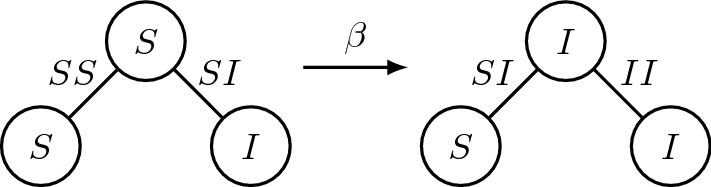
Example of the role of triples. The rightmost edge (of the triple on the left) turns from *SI* to *II* because the infection spreads to the central node; the leftmost edge turns from *SS* to *SI* because it belongs to a triple *SSI*

In order to fully describe the dynamics of the system, we need an ODE for [*SI*] as well. To understand how the number of edges [*SI*] evolve in time, we need to consider the role of triples, as exemplified in Fig. 1. A triple is a path of length 2 through a central node in state *Y*, connected to two nodes in state *X* and *Z*, respectively; we indicate such a triple with *XYZ*. The positions of *X* and *Z* are interchangeable, and the most important node is the central one, as we will explain shortly.

The only change of the system which depends on the presence of a specific edge is the contagion which brings $SI \rightarrow II$. Direct neighbours of a node in the state *S* which get infected, i.e. the node *X* in a triple *XSI*, see their edge *XS* change to *XI* due to their belonging to the triple. The two other possible changes in the system, namely the recovery (a node in state *I* becoming *R*, which happens at a rate $\gamma$) and the loss of immunity (a node in state *R* becoming *S*, which happens at a rate $\epsilon$) only happen at a node level, so the only nodes which see this change are the direct neighbours of the node changing state, and we do not need to consider their belonging to a triple.

**Fig. 2:**
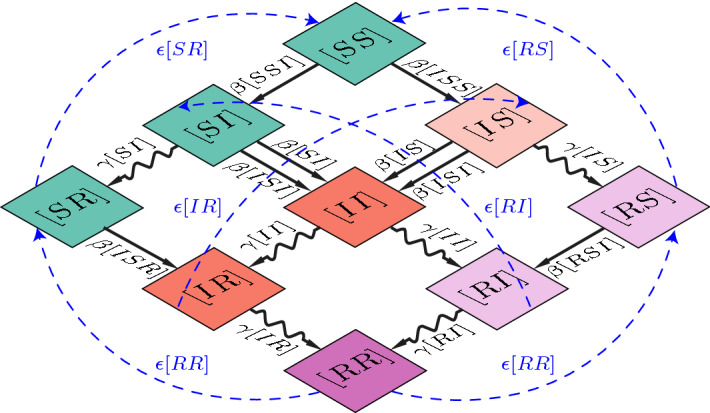
Complete description of the edges dynamics considering edges and triples. Straight lines: infections; wobbly lines: recovery; dashed lines: loss of immunity. The base diagram is the same which appears in Kiss et al. (2017), to visually describe their SIR model; the new, slow dynamics in our model are the dashed blue arrows, symbolizing loss of immunity

For clarity, we fix a lexicographic order $S \prec I \prec R$ for nodes and edges, and write the explicit equations for the edges which follow this order only. If we take into account all the triples with a central node in state *S* and at least one node *I*, which could infect the central one (as described in Fig. 2), we obtain the following system of ODEs, which describes the evolution in time of nodes and edges:

3 $$\begin{aligned} {[S]}'&={}-\beta [SI]+ \epsilon (1-[S]-[I]),\nonumber \\ {[I]}'&={}\beta [SI]- \gamma [I],\nonumber \\ {[SS]}'&={}2\epsilon [SR]-2\beta [SSI],\nonumber \\ {[SI]}'&={}-(\gamma +\beta )[SI]+\epsilon [IR]+\beta [SSI]-\beta [ISI],\nonumber \\ {[SR]}'&={}\gamma [SI] -\epsilon [SR] +\epsilon [RR]- \beta [ISR],\nonumber \\ {[II]}'&={}2\beta [SI]-2\gamma [II]+2\beta [ISI],\nonumber \\ {[IR]}'&={}\gamma [II]-(\gamma +\epsilon ) [IR]+\beta [ISR],\nonumber \\ {[RR]}'&={} 2\gamma [IR]-2\epsilon [RR]. \end{aligned}$$

Notice the 2 which multiplies the right hand sides of edges connecting nodes in the same state: as we mentioned above, they are always counted twice, whether they are created or lost. To fully describe the system, we would then need to have ODEs for triples, quadruples, etc. Instead, we proceed as in Kiss et al. (2017), and apply *moment closures*.

### Moment closures

Moment closure methods are approximation methods used in many contexts, in order to reduce large (or infinite) dimensional systems of equations to a smaller finite dimension (Kuehn 2016). Proceeding as in Kiss et al. (2017, Sec. 4.2), one can approximate the edges as functions of the nodes, or triples as functions of nodes and edges. If we choose the first option, assuming independence between the state of nodes, we can approximate all edges as follows:

4 $$\begin{aligned}{}[XY] \approx n [X][Y]. \end{aligned}$$

This implies that we lose the network structure and, up to rescaling the infection parameter by ${\tilde{\beta }}=n\beta$, we recover the SIRS system already studied in Jardón-Kojakhmetov et al. (2021).

#### Lemma 1

Consider (2). Applying approximation (4) and rescaling ${\tilde{\beta }}=n\beta$, one recovers the *SIRS* system studied in Jardón-Kojakhmetov et al. (2021), which is characterized by an asymptotic stability of the endemic equilibrium for orbits starting in the set $\left\{ (S,I,R)\in {\mathbb {R}}^3_{\ge 0}\, | \, \, S+I+R\le 1, I>0 \right\}$.

Instead, in this work we choose to apply the second order approximation, and hence we approximate each triple with the formula given in equation (4.6) of Kiss et al. (2017), namely

5 $$\begin{aligned}{}[XYZ]\approx \frac{n-1}{n} \frac{[XY][YZ]}{[Y]}. \end{aligned}$$

This approximation is based on the conditional independence between the states of neighbors of a node, using a counting argument, which for clarity we recall from Kiss et al. (2017). The total number of edges starting from a node in state *Y* is *n*[*Y*], while the total number of edges in state *XY* is [*XY*]; this means that a fraction [*XY*]/(*n*[*Y*]) of edges starting from a node in state *Y* reach a node in state *X*. With the same procedure, we obtain a fraction [*YZ*]/(*n*[*Y*]) of edges which connect a node in state *Y*, from which we start, with one in state *Z*. Hence, selecting a node in state *Y* and two of his direct neighbours *u* and *v*, and using the conditional independence of *u* and *v*, the probability of them forming a triple *XYZ* is $[XY][YZ]/(n^2[Y]^2)$. Combinatorics tell us there are $n(n-1)$ ways of picking *u* and *v*, and [*Y*] nodes in state *Y*; multiplying $n(n-1)\cdot [Y]\cdot [XY][YZ]/(n^2[Y]^2)$, we obtain formula (5).

## Analysis of the model

In this section we present the pair approximation SIRS model, and give our main analytical results. First, we are going to reduce the dimension of the system, exploiting three conserved quantities. Secondly, we introduce a formulation for the basic reproduction number for the system, and we describe the behaviour of the fast limit system. Next, we are going to derive the equilibria of the system in the biologically relevant region, and we show that the slow manifold of our perturbed system is exponentially close to the critical manifold. Lastly, we rescale the system in an ${\mathcal {O}}(\epsilon )$-neighbourhood of the critical manifold, with a scaling similar to the one proposed in Jardón-Kojakhmetov et al. (2021), and we apply the entry–exit formulation.

Throughout the analysis, we notice that the parabola $[SS]=n[S]^2$, i.e. approximation (4) applied to the edges in state [*SS*], on the critical manifold is of particular importance for the dynamics.

### Fast–slow model

In this section, we derive the system we will study for the remainder of the article, applying moment closure to (3) and reducing its dimension.

Applying approximation (5) to every triple in system (3), we obtain the following singularly perturbed autonomous system in non-standard form:

6 $$\begin{aligned} {[S]}'&={}-\beta [SI]+ \epsilon (1-[S]-[I]),\nonumber \\ {[I]}'&={}\beta [SI]- \gamma [I],\nonumber \\ {[SS]}'&={}2\epsilon [SR]-2\beta \frac{n-1}{n}\frac{[SS][SI]}{[S]},\nonumber \\ {[SI]}'&={}-(\gamma +\beta )[SI]+\epsilon {[IR]}+\beta \frac{n-1}{n}[SI]\left( \frac{[SS]}{[S]}-\frac{[SI]}{[S]}\right) ,\nonumber \\ {[SR]}'&={}\gamma [SI] -\epsilon [SR] +\epsilon [RR]- \beta \frac{n-1}{n}\frac{[SI][SR]}{[S]},\nonumber \\ {[II]}'&={}2\beta [SI]-2\gamma [II]+2\beta \frac{n-1}{n} \frac{[SI]^2}{[S]},\nonumber \\ {[IR]}'&={}\gamma [II]-(\gamma +\epsilon ) [IR]+\beta \frac{n-1}{n}\frac{[SI][SR]}{[S]},\nonumber \\ {[RR]}'&={} 2\gamma [IR]-2\epsilon [RR], \end{aligned}$$

in which, as from our assumptions, the processes of infection and recovery are fast, and the process of loss of immunity is slow. By construction, the sum of all the edges starting from a node in the state [*S*] (or [*I*] or [*R*], respectively) is equal to

7a $$\begin{aligned}{}[SS]+[SI]+[SR]&=n[S], \end{aligned}$$

7b $$\begin{aligned} +[II]+[IR]&=n[I], \end{aligned}$$

7c $$\begin{aligned} +[IR]+[RR]&=n[R], \end{aligned}$$

which allows us to remove the equation governing [*SR*] (and [*IR*] and [*RR*], respectively). This can be checked by carefully computing the difference of the derivatives of the right hand side(s) and the left hand side(s) of (7). By doing so, we reduce the dimension of the system, obtaining

8a $$\begin{aligned}{}[S]'&={}-\beta [SI]+ \epsilon (1-[S]-[I]), \end{aligned}$$

8b $$\begin{aligned} '&={}\beta [SI]- \gamma [I], \end{aligned}$$

8c $$\begin{aligned} '&={}2\epsilon (n[S]-[SS]-[SI])-2\beta \frac{n-1}{n}\frac{[SS][SI]}{[S]}, \end{aligned}$$

8d $$\begin{aligned} '&={}-(\gamma +\beta )[SI]+\epsilon (n[I]-[SI]-[II])+\beta \frac{n-1}{n}[SI] \left( \frac{[SS]}{[S]}-\frac{[SI]}{[S]}\right) , \end{aligned}$$

8e $$\begin{aligned} '&={}2\beta [SI]-2\gamma [II]+2\beta \frac{n-1}{n} \frac{[SI]^2}{[S]}. \end{aligned}$$

The *basic reproduction number* $R_0$ can be obtained (Kiss et al. (2017, p. 140)) for the limit as $\epsilon \rightarrow 0$ of system (8) as

9 $$\begin{aligned} R_0 =\frac{\beta (n-2)}{\gamma }. \end{aligned}$$

We notice that, for (9) to be well-defined and dependent on the parameters of the system, we need $n>2$. The equality $n=2$ describes the very special case of a ring network, i.e., a connected network in which all nodes have exactly two neighbours. In the remainder of the paper we assume $R_0>1$ and $n>2$.

#### Remark 1

We notice that the threshold $R_0 \lessgtr 1$ in (9) is equivalent to

10 $$\begin{aligned} R_1:=\frac{\beta (n-1)}{\beta +\gamma }\lessgtr 1 \iff R_2:=\frac{\beta n}{2\beta +\gamma }\lessgtr 1, \end{aligned}$$

since they all correspond to $\beta (n-2)\lessgtr \gamma$. A formula corresponding to $R_1$ is given in Kiss et al. (2017), shortly after the definition of $R_0$.

We notice that $R_1$ has a much more intuitive biological interpretation than $R_0$. Consider a network with all the nodes in susceptible state *S*, except one in state *I*. Consider one of the *n* edges in state *IS*: this could either transition to *RS*, at a rate $\gamma$, and the epidemics would die out immediately, or spread the infection to the node in state *S*, at a rate $\beta$, and become an edge *II*. If the latter happens, with probability $\beta /(\beta +\gamma )$, $(n-1)$ new edges move to state *SI*; hence, $R_1$ can be interpreted in the classical meaning of “the number of edges infections caused by one infected edge in an otherwise susceptible population”. Recall that the disease spreads only through edges *SI* (or *IS*, equivalently), so their number should be the quantity we measure in order to quantify the contagiousness of the disease; an edge *II* can not be used to spread the disease.

Now we compute the basic reproduction number $R_1$ for system (8) and $\epsilon >0$ sufficiently small.

#### Proposition 1

The basic reproduction number $R_1$ for system (8) is given by

11 $$\begin{aligned} R_1=\frac{\beta (n-1)(\gamma +\epsilon )}{\gamma (\gamma +\beta +\epsilon )}. \end{aligned}$$

#### Proof

We use the method first introduced in Diekmann et al. (1990), and then generalized in Van den Driessche and Watmough (2002) (see also Diekmann et al. (2010)). We linearize system (6) at the disease free equilibrium

$$\begin{aligned} ([S],[I],[SS],[SI],[SR],[II],[IR],[RR])=(1,0,n,0,0,0,0,0) \end{aligned}$$

focusing on the infected compartments. In this case we choose as variables describing the infected compartments [*SI*], [*II*]/2 and [*IR*] obtaining

$$\begin{aligned} \begin{pmatrix} [SI]\\ [II]/2\\ [IR] \end{pmatrix}' =A \begin{pmatrix} [SI]\\ [II]/2\\ [IR] \end{pmatrix}, \end{aligned}$$

with the matrix *A* given by

$$\begin{aligned} A= \begin{pmatrix} \beta (n-2)-\gamma &{} 0 &{} \epsilon \\ \beta &{} -2\gamma &{} 0\\ 0 &{} 2\gamma &{} -(\gamma +\epsilon ) \end{pmatrix}. \end{aligned}$$

We split $A=M-V$, with *V* invertible, *M* and $V^{-1}$ having non-negative entries. There are clearly many ways of doing that, but the preferred splitting is such that *M* and *V* can be interpreted as the *transmission* (i.e. relative to new infections) and *transition* matrix (i.e. relative to any other change of state), respectively. Then, we compute

$$\begin{aligned} R_1 = \rho (M V^{-1}), \end{aligned}$$

where $\rho$ indicates the spectral radius of a matrix. The choice for the two matrices is

$$\begin{aligned} M= \begin{pmatrix} \beta (n-1) &{} 0 &{} 0\\ 0 &{} 0 &{} 0\\ 0 &{} 0 &{} 0 \end{pmatrix}, \quad V= \begin{pmatrix} \gamma +\beta &{} 0 &{}-\epsilon \\ -\beta &{} 2\gamma &{} 0\\ 0 &{} -2\gamma &{} \gamma +\epsilon \end{pmatrix}. \end{aligned}$$

It can easily be checked, then, that $V^{-1}$ has non-negative entries, and that, since $M V^{-1}$ has two rows of zeros,

12 $$\begin{aligned} \rho (M V^{-1})=(M V^{-1})_{1,1}= R_1:=\frac{\beta (n-1)(\gamma +\epsilon )}{\gamma (\gamma +\beta +\epsilon )}. \end{aligned}$$

This finishes the proof. $\square$

#### Remark 2

The perturbed $R_1$ given in (11) has a similar biological interpretation for the perturbed system to the one given for the corresponding $R_1$ (10) of the limit system as $\epsilon \rightarrow 0$. We need to compute $R_1$, which corresponds to the average number of *SI* edges produced by an *SI* edge in a totally susceptible population. As in the previous case, an edge *SI* will become an edge *II* with probability $\beta /(\beta +\gamma )$, producing in this case $n-1$ edges *SI*. However, the original edge *II*, after having become *IR* can become again an *IS* edge with probability $\epsilon /(\epsilon +\gamma )$. After having returned to the state *SI*, the edge will produce other $R_1$ *SI* edges, since the pairwise model does not consider higher order correlation and does not “remember” that the neighbours of *S* had already been infected once. Hence

$$\begin{aligned} R_1 = \frac{\beta }{ \beta +\gamma }\left( n-1 + \frac{\epsilon }{\epsilon + \gamma } R_1\right) , \end{aligned}$$

from which one obtains (12). Through this argument, we see that threshold for the SIRS model is different from the one for the SIR model, while in the homogeneous mixing case the two coincide.

#### Lemma 2

System (8) is well-posed in the convex set

13 $$\begin{aligned}&\Delta = \left\{ ([S],[I],[SS],[SI],[II])\in {\mathbb {R}}^5_{\ge 0} | \right. \nonumber \\&\quad \left. 0 \le [S]+[I] \le 1 \right\} \cap \left\{ 0 \le [SS]+[SI] \le n[S], 0 \le [SI]+[II]\le n[I] \right\} .\nonumber \\ \end{aligned}$$

The set is forward invariant under the flow of (8), for $\epsilon \ge 0$, so that solutions of (8) are global in time.

#### Proof

Apparently the right-hand side of (8) has a singularity at $[S] = 0$; however, in the set $\Delta$, the terms [*SI*]/[*S*] and [*SS*]/[*S*] are both bounded by *n*, so that the right-hand side is indeed Lipschitz. Hence, system (8) has a local solution. Furthermore, it can be easily checked that the system is forward invariant by showing that the flow is pointing inwards on the boundary of $\Delta$. Hence, solutions of system (8) are global in time. $\square$

### Fast limit

In this section, we study the fast subsystem (or layer equations) corresponding to the limit of system (8) as $\epsilon \rightarrow 0$ on the fast time scale. Hence, taking the limit $\epsilon \rightarrow 0$ in system (8), we obtain

14a $$\begin{aligned}{}[S]'&={}-\beta [SI], \end{aligned}$$

14b $$\begin{aligned} '&={}\beta [SI]- \gamma [I], \end{aligned}$$

14c $$\begin{aligned} '&={}-2\beta \frac{n-1}{n}\frac{[SS][SI]}{[S]}, \end{aligned}$$

14d $$\begin{aligned} '&={}-(\gamma +\beta )[SI]+\beta \frac{n-1}{n}[SI]\left( \frac{[SS]}{[S]}-\frac{[SI]}{[S]}\right) , \end{aligned}$$

14e $$\begin{aligned} '&={}2\beta [SI]-2\gamma [II]+2\beta \frac{n-1}{n} \frac{[SI]^2}{[S]}. \end{aligned}$$

For ease of notation, we introduce

$$\begin{aligned} \begin{aligned} {[\cdot ]}_0&={}[\cdot ](0),\\ {[\cdot ]}_{\infty }&={}\lim _{t \rightarrow +\infty }[\cdot ](t).\\ \end{aligned} \end{aligned}$$

In the fast dynamics, the susceptible population can only decrease, and eventually the infected population will not have any more susceptibles to “recruit” and will decrease as well. In particular, we prove the following:

#### Proposition 2

Consider system (14); the states [*S*] and [*SS*] are decreasing for all $t\ge 0$, and they tend asymptotically to positive constants $[S]_{\infty }$ and $[SS]_{\infty }$. The variables [*I*], [*SI*], [*II*] and [*IR*] all have the limit $[I]_{\infty }=[SI]_{\infty }=[II]_{\infty }=[IR]_{\infty }=0$.

#### Proof

We proceed to show the claims of the proposition: for [*SS*] (and implicitly for [*SR*], referring to (7a)), we give the limit value as a function of $[S]_\infty$, $[S]_0$ and $[SS]_0$. We introduce the auxiliary variables $u:=\frac{[SI]}{[S]}$ and $v:=\frac{[SS]}{[S]}$. From (14a) and (14d) we see that

$$\begin{aligned} u'=-(\gamma +\beta )u+\beta u\left( \frac{n-1}{n}v+\frac{1}{n}u\right) , \end{aligned}$$

while from (14a) and (14c) we see that

15 $$\begin{aligned} v'=-\beta \frac{n-2}{n}uv. \end{aligned}$$

From our analysis, for any initial point we have $0\le [SS]+[SI]\le n [S]$ and $[SS],[SI]\ge 0$. This implies that, for all times

$$\begin{aligned} u\ge 0, \quad v\ge 0, \quad u+v\le n. \end{aligned}$$

Note that from (15) *v* is clearly decreasing for $n>2$, and we see that

16 $$\begin{aligned} u'+v'=-\gamma u -\beta u \left( 1-\frac{u+v}{n}\right) <0. \end{aligned}$$

Recall Lemma 2, which implies $v\ge 0$; if $v=0$, then $[SS]=0$, and from Eq. (14c) we observe that [*SS*] will not change, so 0 is its corresponding limit value. Assume then $v>0$: since $v'<0$, $v \rightarrow v_\infty$ monotonically as $t \rightarrow \infty$, and since $0\le u+v\le n$, this implies that $u \rightarrow u_\infty$ as $t \rightarrow \infty$ as well. Then we notice that

17 $$\begin{aligned} 0<-\int _{0}^{\infty }(u'(z)+v'(z))~\text {d}z=u_0+v_0- u_\infty - v_\infty <\infty . \end{aligned}$$

We notice that we can rewrite (17) using (16) and obtain

18 $$\begin{aligned} \infty> & {} -\int _{0}^{+\infty }(u'(z)+v'(z))\text {d}z=\int _{0}^{+\infty }\left( \gamma u(z) +\beta u(z) \left( 1-\frac{1}{n}(u(z)+v(z))\right) \right) ~\text {d}z\nonumber \\ \end{aligned}$$

19 $$\begin{aligned}> & {} \gamma \int _{0}^{+\infty }u(z)~\text {d}z. \end{aligned}$$

This means that

20 $$\begin{aligned} \int _{0}^{+\infty }u(z) \text {d}z<+\infty \implies u_\infty =0, \end{aligned}$$

which implies, recalling that $[SI]=u[S]$ and $[S]_\infty <\infty$, that $[SI]_\infty =0$. We can now rewrite (14a) as

$$\begin{aligned}{}[S]'=-\beta u [S], \end{aligned}$$

which implies

21 $$\begin{aligned}{}[S]_\infty = [S]_0 \exp \left( -\beta \int _{0}^{+\infty }u(z) \text {d}z\right) >0. \end{aligned}$$

Similarly, using (15), we can show that

22 $$\begin{aligned} v_\infty = v_0 \exp \left( -\beta \frac{n-2}{n} \int _{0}^{+\infty }u(z) \text {d}z\right) >0, \end{aligned}$$

which implies, using (20) and (21), and recalling that $[SS]=v[S]$, that $[SS]_\infty >0$. In particular, combining (21) and (22), we can write

23 $$\begin{aligned}{}[SS]_\infty = [SS]_0 \left( \frac{[S]_\infty }{[S]_0}\right) ^{\frac{2n-2}{n}}. \end{aligned}$$

We notice, from (7a), that this implies that [*SR*] converges to a non-negative limit as well. Combining (14a) and (14b) as above, we show that [*I*] vanishes as $t\rightarrow \infty$ as well:

$$\begin{aligned}{}[S]'+[I]'=-\gamma [I]<0. \end{aligned}$$

Since $[S]\rightarrow [S]_\infty$ as $t\rightarrow +\infty$, also $[I]\rightarrow [I]_\infty$. Proceeding as in (19), it can be shown that $[I]_\infty =0$. This yields, by (7b), that $[II]_\infty =0$ and $[IR]_\infty =0$. $\square$

#### Remark 3

A relation between [*SS*](*t*) and [*S*](*t*) for system (14) analogous to (23) holds for all *t*. Indeed, noticing that

$$\begin{aligned} V(t)=\ln ([SS](t))-2\frac{n-1}{n} \ln ([S](t)), \end{aligned}$$

is a constant of motion for system (14), we observe that for any $t\ge 0$ the relation

24 $$\begin{aligned}{}[SS](t)=[SS]_0 \left( \frac{[S](t)}{[S]_0}\right) ^{\frac{2n-2}{n}}, \end{aligned}$$

holds.

The equilibria of the limit system are all of the form $[S]=S^* \in [0,1]$, $[I]=0$, $[R]=1-S^*$; $[SS]=SS^* \ge 0$, $[SI]=0$, $[SR]=SR^*\ge 0$, $[II]=0$, $[IR]=0$, $[RR]=RR^*\ge 0$ with $SS^* + SR^* =nS^*$ and $SR^* + RR^* =n(1-S^*)$; i.e., they lie on the critical manifold (25).

The eigenvalues of the linearization of system (8) on the critical manifold

25 $$\begin{aligned} {\mathcal {C}}_0:=\left\{ ([S],[I],[SS],[SI],[II])\in {\mathbb {R}}^5_{\ge 0} | [I]=[SI]=[II]=0\right\} , \end{aligned}$$

are

$$\begin{aligned} \lambda _1=\lambda _2=0, \end{aligned}$$

corresponding to the slow variables [*S*] and [*SS*],

$$\begin{aligned} \lambda _3=\frac{\lambda _4}{2}=-\gamma <0, \end{aligned}$$

and

26 $$\begin{aligned} \lambda _5=\beta \frac{(n-1)[SS]}{n[S]}-(\gamma +\beta ) . \end{aligned}$$

In particular, $\lambda _5$ changes sign on the hyperplane defined by $\beta (n-1)[SS]-n(\gamma +\beta )[S]=0$. We notice that $\beta (n-1)>0$, since we assume $n>2$. Considering (26), we define the *loss of hyperbolicity line* on the critical manifold ${\mathcal {C}}_0$ as

27 $$\begin{aligned}{}[SS]=\frac{n(\beta +\gamma )}{\beta (n-1)}[S]=:L[S]. \end{aligned}$$

We now give a closed formula for the value of $[S]_\infty$.

#### Proposition 3

Consider a generic initial condition $([S]_0,[SS]_0)$ in the repelling region of ${\mathcal {C}}_0$, i.e. satisfying $[SS]_0>L[S]_0$, and initial conditions for $[SI](0)={\mathcal {O}}(\epsilon )$. The limit value under the fast flow $[S]_\infty$ is ${\mathcal {O}}(\epsilon )$ close to the unique zero smaller than $[S]_0$ of the function

28 $$\begin{aligned} H(x)=n\frac{\beta +\gamma }{\beta } \left( x^{\frac{1}{n}}-[S]_0^{\frac{1}{n}}\right) -[SS]_0\left( [S]_0^{\frac{2}{n}-2}x^{1-\frac{1}{n}}-[S]_0^{\frac{1}{n}-1}\right) . \end{aligned}$$

#### Proof

We proceed as in (Bidari et al. 2016, Sec. 3). From our assumptions, $[SI](0)={\mathcal {O}}(\epsilon )$. Combining (14a), (14d) and (24), we obtain

$$\begin{aligned}{}[SI]'-\frac{n-1}{n}\frac{[SI]}{[S]}[S]'= \frac{\beta +\gamma }{\beta }[S]' -\frac{n-1}{n}[SS]_0[S]_0^{\frac{2}{n}-2}[S]^{\frac{n-2}{n}}[S]'. \end{aligned}$$

Multiplying both sides by the integrating factor $[S]^{\frac{1-n}{n}}$ we get

29 $$\begin{aligned} \frac{\text {d}}{\text {d}t}\left( [SI][S]^{\frac{1-n}{n}} \right) =\frac{\beta +\gamma }{\beta }[S]^{\frac{1-n}{n}}[S]' -\frac{n-1}{n}[SS]_0[S]_0^{\frac{2}{n}-2}[S]^{-\frac{1}{n}}[S]'. \end{aligned}$$

Integrating (29) from $t=0$ to $t=+\infty$, and recalling that, by Proposition 2, $[SI]_\infty =0$, we obtain

30 $$\begin{aligned} -[SI](0)[S]_0^{\frac{1-n}{n}}=n\frac{\beta +\gamma }{\beta } [S]^{\frac{1}{n}} \bigg |_{t=0}^{+\infty }-[SS]_0[S]_0^{\frac{2}{n}-2}[S]^{\frac{n-1}{n}} \bigg |_{t=0}^{+\infty }. \end{aligned}$$

Since, by assumption, the left-hand side of (30) is ${\mathcal {O}}(\epsilon )$, we ignore it, and we consider the right-hand side only. Hence, we find $[S]_\infty$ by solving

$$\begin{aligned} n\frac{\beta +\gamma }{\beta } [S]^{\frac{1}{n}} \bigg |_{t=0}^{+\infty }=[SS]_0[S]_0^{\frac{2}{n}-2}[S]^{\frac{n-1}{n}} \bigg |_{t=0}^{+\infty }, \end{aligned}$$

from which we immediately obtain that $[S]_\infty$ is given as a zero of the function *H*(*x*) defined in (28). Next, we prove that such a zero is unique.

Recall that $\frac{[SS]_0}{[S]_0}\le n$. Therefore, we have

$$\begin{aligned} H(0)=[S]_0^{\frac{1}{n}}\left( -n\frac{\beta +\gamma }{\beta }+\frac{[SS]_0}{[S]_0}\right) <0, \quad H([S]_0)=0. \end{aligned}$$

Moreover,

$$\begin{aligned}&H'(x)=\frac{\gamma +\beta }{\beta }x^{\frac{1}{n}-1} -\frac{n-1}{n}[SS]_0[S]_0^{\frac{2}{n}-2}x^{-\frac{1}{n}}\\&=x^{\frac{1}{n}-1}\left( \frac{\gamma +\beta }{\beta }- \frac{n-1}{n}[SS]_0[S]_0^{\frac{2}{n}-2}x^{1-\frac{2}{n}} \right) . \end{aligned}$$

From (27) we see that $H'(x)>0$ for

$$\begin{aligned} x<\left( \frac{L[S]_0}{[SS]_0} \right) ^{\frac{n}{n-2}}[S]_0=:[S]_*\left( [S]_0,[SS]_0\right) . \end{aligned}$$

Clearly, $[S]_*=[S]_*([S]_0,[SS]_0)<[S]_0$, since we assumed $[SS]_0>L[S]_0$. Lastly,

$$\begin{aligned} H'([S]_0)=[S]_0^{\frac{1}{n}-1}\left( \frac{\gamma +\beta }{\beta } - \frac{n-1}{n} \frac{[SS]_0}{[S]_0}\right) <0 \quad \hbox { if }\quad [SS]_0>L[S]_0. \end{aligned}$$

Hence, *H*(*x*) increases on the interval $$[0,[S]_*)$$, has a positive maximum in $$x=[S]_*$$, and then decreases towards 0; in particular, it has a unique zero on the interval $$[0,[S]_*)$$, and hence in the interval $$[0,[S]_0)$$. $$\square $$

#### Remark 4

**Fig. 3:**
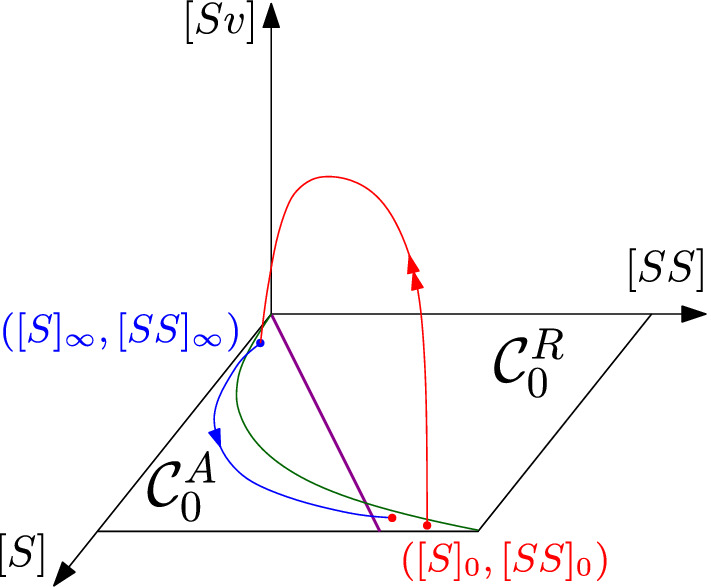
Red curve: evolution of the point $([S]_0,[SS]_0)$ under the fast flow. Blue curve: evolution of the point $([S]_\infty ,[SS]_\infty )$ under the slow flow. Green curve: curve $[SS]=\alpha ([S])$ defined in (39). Purple line: line of loss of hyperbolicity $[SS]=L[S]$ of the critical manifold of system (8), which divides the attracting region ${\mathcal {C}}_0^A$ and the repelling one ${\mathcal {C}}_0^R$ (color figure online)

Recall (27) and Proposition 2. Given a pair $([S]_0,[SS]_0)$ in the repelling region ${\mathcal {C}}_0^R$ above the line $[SS]=L[S]$ (i.e., where $\lambda _5>0$), its image under the fast flow (14), approximated up to ${\mathcal {O}}(\epsilon )$ by formulas (28) and (23), is in the attracting region ${\mathcal {C}}_0^A$ below the line $[SS]=L[S]$ (i.e., where $\lambda _5<0$); refer to Fig. 3 for a visualization.

#### Remark 5

Recall (9), and that we assume $R_1>1$. Then $L=\frac{n(\beta +\gamma )}{\beta (n-1)}=\frac{n}{R_1}<n$. Hence, the purple line $[SS]=L[S]$ in Fig. 3 is always below the line $[SS]=n[S]$.

#### Remark 6

The loss of hyperbolicity line can be rewritten as $R_1[SS]/(n[S])=1$, meaning there is a minimum ratio of susceptible edges to susceptible nodes for the epidemic to “explode”. In this form, the line can more straightforwardly be related to the threshold $R_0 S =1$ in the classic *SIR* model. Moreover, since by (7a) we derive the constraint $[SS]\le n[S]$, if $R_1<1$ the epidemic can never start, as the loss of hyperbolicity in that case lies in an unreachable region of the critical manifold.

### Equilibria of the perturbed system

The following proposition discusses the equilibria of system (8).

#### Proposition 4

For $\epsilon > 0$ sufficiently small and $R_0 > 1$, system (8) has 2 equilibria in the relevant region of ${\mathbb {R}}^5$.

**Disease free equilibrium:**

$$\begin{aligned}{}[S]=1, \quad [I]=0, \quad [SS]=n, \quad [SI]=0, \quad [II]=0. \end{aligned}$$

**Endemic equilibrium:** to their first order on $\epsilon$ the components are given by:

31 $$\begin{aligned} {[S]}= & {} \frac{(n-1)(\gamma +\beta )}{\left( n^2-n-1\right) \beta - \gamma } +{\mathcal {O}}(\epsilon ), \nonumber \\ {[I]}= & {} \epsilon \frac{n ((n-2) \beta -\gamma )}{\gamma \left( \left( n^2-n-1\right) \beta -\gamma \right) }+{\mathcal {O}}(\epsilon ^2), \nonumber \\ {[SS]}= & {} \frac{n (\gamma +\beta )^2}{\beta ( \left( n^2-n-1\right) \beta - \gamma )}+{\mathcal {O}}(\epsilon ),\nonumber \\ {[SI]}= & {} \epsilon \frac{n ((n-2)\beta -\gamma )}{\beta \left( \left( n^2-n-1\right) \beta -\gamma \right) }+{\mathcal {O}}(\epsilon ^2), \nonumber \\ {[II]}= & {} \epsilon \frac{n ((n-2) \beta -\gamma )}{\gamma \left( \left( n^2-n-1\right) \beta -\gamma \right) }+{\mathcal {O}}(\epsilon ^2). \end{aligned}$$

#### Proof

The disease free equilibrium is trivial. The endemic equilibrium is computed by expanding the variables in power series of $\epsilon$, e.g. $[S]=S_0+\epsilon S_1 + {\mathcal {O}}(\epsilon ^2)$, substituting them in system (8), equating the right-hand sides to 0 and matching powers of $\epsilon$. $\square$

#### Remark 7

Since we assume $R_0=\frac{\beta (n-2)}{\gamma }>1$, recall Remark 1 and (11), the numerators of [*I*], [*SI*] and [*II*] of (31), as well as all the denominators, are strictly positive for $\epsilon >0$ small enough.

We notice that the disease free equilibrium belongs to ${\mathcal {C}}_0$ defined in (25), and by computing the corresponding $\lambda _5 = \beta (n-2)-\gamma =\gamma (R_0-1)-{\mathcal {O}}(\epsilon )>0$, we show that it is unstable. Moreover, we notice that the endemic equilibrium is ${\mathcal {O}}(\epsilon )$ close to the line $[SS]=L[S]$ defined in (27); hence, it approaches it as $\epsilon \rightarrow 0$. See also Lemma 5 below.

### Slow manifold

In this section we provide a multiple time scale description of the disease-free, or near disease-free states.

#### Proposition 5

The slow manifold of system (8) is exponentially close in $\epsilon$ to the critical manifold ${\mathcal {C}}_0$ given by (25).

#### Proof

The invariant manifold ${\mathcal {C}}_0$ is an invariant manifold also for system (8) with $\epsilon >0$: by direct substitution, we have that $[I]'$, $[SI]'$ and $[II]'$ are zero on ${\mathcal {C}}_0$. Hence, ${\mathcal {C}}_0$ is invariant and satisfies all the conclusions of Fenichel’s theorem, and so it is one possible slow manifold. By Fenichel’s theorem, all slow manifolds are exponentially close to each other in the normally hyperbolic region; invariance allows us to extend at least one slow manifold across the line where we do not have normal hyperbolicity, namely ${\mathcal {C}}_0$. $\square$

We provide an explicit computation of the slow manifold, expanding it in orders of $\epsilon$, in Appendix A.

The slow dynamics on the slow manifold $[I]=[SI]=[II]=0$ are given by:

$$\begin{aligned} \begin{aligned} {[S]}'&={} \epsilon (1-[S]),\\ {[SS]}'&={}2\epsilon (n[S]-[SS]), \end{aligned} \end{aligned}$$

which, rescaling the system to the slow time variable $\tau =\epsilon t$, becomes

32 $$\begin{aligned} \begin{aligned} {\dot{[S]}}&={} 1-[S],\\ {\dot{[SS]}}&={} 2 (n[S]-[SS]). \end{aligned} \end{aligned}$$

Recall that $[S]_\infty$ and $[SS]_\infty$ are the initial conditions for the slow flow. Solving (32) explicitly yields

33 $$\begin{aligned} \begin{aligned} {[S]}(\tau )&={} ([S]_\infty -1)e^{- \tau }+1,\\ {[SS]}(\tau )&={} 2 ([S]_\infty -1)n e^{-2\tau } (e^{ \tau }-1)+([SS]_\infty -n)e^{-2 \tau }+n, \end{aligned} \end{aligned}$$

meaning that $[S]\rightarrow 1$, $[SS]\rightarrow n$ exponentially fast, as we would expect, since in the slow dynamics, on the node level, the variable [*R*] can only decrease, and [*S*] can only increase.

For its importance in the dynamics, we introduce the following notation

34 $$\begin{aligned} \Gamma := \{ ([S],[SS])\in [0,1]\times [0,n]| [SS]=n[S]^2 \}. \end{aligned}$$

#### Lemma 3

The parabola $\Gamma$, given by (34), is uniformly attracting for the slow reduced subsystem (32).

#### Proof

Recall (33). The distance between a solution curve of (33) and $\Gamma$ can be parametrized by the slow time $\tau$. With this in mind, let $d(\tau )$ denote such distance, we then have

$$\begin{aligned} \begin{aligned} d(\tau )&=|[SS](\tau )-n[S]^2(\tau )|\\&=|2n \left( [S]_\infty -1\right) e^{- \tau } -2n \left( [S]_\infty -1\right) e^{-2\tau }+\left( [SS]_\infty -n\right) e^{-2 \tau }\\&\quad +n-n\left( [S]_\infty -1\right) ^2 e^{-2\tau }-n-2n\left( [S]_\infty -1\right) e^{-\tau }|\\&=|e^{-2\tau }\left( [SS]_\infty -n[S]_\infty ^2\right) |\\&=e^{-2\tau }d(0), \end{aligned} \end{aligned}$$

which means that an orbit starting in any point $([S]_\infty , [SS]_\infty )\in (0,1)\times (0,n)$ approaches exponentially fast the parabola $\Gamma$ (34). $\square$

#### Lemma 4

Consider an orbit starting (i.e. exiting the slow manifold) ${\mathcal {O}}(\delta _2)$, where $0<\delta _2 \ll 1$, away from the parabola $[SS]=n[S]^2$, in a point with $[S](0)=[S]_0$ in the repelling region of ${\mathcal {C}}_0$, i.e. satisfying $[SS]_0>L[S]_0$. Its limit value under the fast flow $[S]_\infty$ is ${\mathcal {O}}(\delta _2)$ close to the unique zero smaller than $[S]_0$ of

35 $$\begin{aligned} G(x) =\frac{\beta +\gamma }{\beta }\left( x^{\frac{1}{n}}-[S]_0^{\frac{1}{n}}\right) - [S]_0^{\frac{2}{n}}x^{1-\frac{1}{n}}+[S]_0^{1+\frac{1}{n}}. \end{aligned}$$

#### Proof

Notice that, considering Lemma 3, the assumption of starting close to the parabola is not restrictive. The derivation of *G*(*x*) is analogous to the derivation of *H*(*x*) of Proposition 3, using

$$\begin{aligned}{}[SS](t)=n[S]_0^{\frac{2}{n}}([S](t))^{\frac{2n-2}{n}} \end{aligned}$$

instead of (24), since we assume $[SS]_0=n[S]_0^2$. The uniqueness of the zero is obtained applying Proposition 3 to this specific initial condition. $\square$

**Fig. 4:**
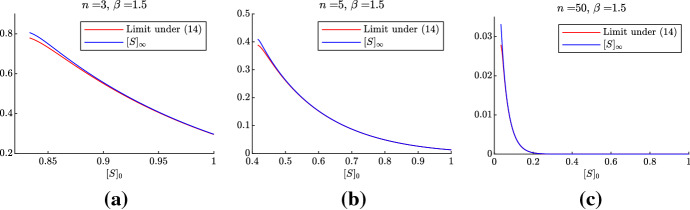
Comparison of the limit value of [*S*] as $t\rightarrow \infty$ of system (14) (red) and formula (35) (blue). We set $[I]_0=[SI]_0=[II]_0=0.001$, $\gamma =1$. With the values of the parameters of (a) (respectively, (b) and (c)), $1/R_1\approx 0.833$ (resp., 0.417 and 0.034), and we only consider values of $[S]_0\ge 1/R_1$, for which the epidemics can start (color figure online)

In Fig. 4 we compare formula (35) and direct integration of the layer system (14) starting with a small fraction of infected nodes.

#### Remark 8

Recall (23). Since we showed that the parabola $\Gamma$ (34) is attracting for the slow flow, we can assume that, after the first slow piece of any orbit, $[SS]_0 = n[S]_0^2 +{\mathcal {O}}(\delta _1)$, where $0<\delta _1 \ll 1$. We can then rewrite (23) as

$$\begin{aligned}{}[SS]_\infty = [SS]_0 \left( \frac{[S]_\infty }{[S]_0}\right) ^{\frac{2n-2}{n}}\approx n [S]_0^2 \left( \frac{[S]_\infty }{[S]_0}\right) ^{\frac{2n-2}{n}}=n [S]_\infty ^2 \left( \frac{[S]_\infty }{[S]_0}\right) ^{-\frac{2}{n}}, \end{aligned}$$

where the $\approx$ symbol indicates an ${\mathcal {O}}(\delta _1)$ error. For *n* large enough, the last factor is close to 1, and the entry point for the slow flow is approximately on the parabola.

### Rescaling

From now on, we are going to assume $n ={\mathcal {O}}(1)$. As we showed in Sect. 3.2, under the fast flow eventually [*I*], [*SI*] and [*II*] will be ${\mathcal {O}}(\epsilon )$; recall (7b), from which we see that $[I]={\mathcal {O}}(\epsilon )$ implies $[SI],[II],[IR]={\mathcal {O}}(\epsilon )$. Proceeding as in Jardón-Kojakhmetov et al. (2021), we rescale $[I]=\epsilon [v]$. This implies, using (7b), that

$$\begin{aligned}{}[SI]= \epsilon [Sv], \quad [II]=\epsilon [vv]. \end{aligned}$$

This rescaling brings the model, after rearranging the variables, to a singularly perturbed system of ODEs, namely

36 $$\begin{aligned} \begin{aligned} {[S]}'&={}-\epsilon \beta [Sv]+ \epsilon (1-[S]-\epsilon [v]),\\ {[SS]}'&={}2\epsilon (n[S]-[SS]-\epsilon [Sv])-2\epsilon \beta \frac{n-1}{n}\frac{[SS][Sv]}{[S]},\\ \epsilon [v]'&={}\epsilon \beta [Sv]- \epsilon \gamma [v],\\ \epsilon [Sv]'&={}-\epsilon (\gamma +\beta )[Sv]+\epsilon ^2 (n[v]-[Sv]-[vv])\\&\quad +\epsilon \beta \frac{n-1}{n}[Sv]\left( \frac{[SS]}{[S]}-\epsilon \frac{[Sv]}{[S]}\right) ,\\ \epsilon [vv]'&={}2\epsilon \beta [Sv]-2\epsilon \gamma [vv]+\epsilon ^2 \beta \frac{n-1}{n} \frac{[Sv]^2}{[S]}, \end{aligned} \end{aligned}$$

which can be rewritten in a standard form, and rescaled to the slow time scale, denoting now the time derivative with respect to the slow time parameter with an overdot, giving

37 $$\begin{aligned} \begin{aligned} {\dot{[S]}}&={}- \beta [Sv]+ (1-[S]-\epsilon [v]),\\ {\dot{[SS]}}&={}2 (n[S]-[SS]-\epsilon [Sv])-2 \beta \frac{n-1}{n}\frac{[SS][Sv]}{[S]},\\ \epsilon \dot{[v]}&={} \beta [Sv]- \gamma [v],\\ \epsilon \dot{[Sv]}&={}-(\gamma +\beta )[Sv]+\epsilon (n[v]-[Sv]-[vv])+ \beta \frac{n-1}{n}[Sv]\left( \frac{[SS]}{[S]}-\epsilon \frac{[Sv]}{[S]}\right) ,\\ \epsilon \dot{[vv]}&={}2\beta [Sv]-2 \gamma [vv]+\epsilon \beta \frac{n-1}{n} \frac{[Sv]^2}{[S]}. \end{aligned} \end{aligned}$$

Taking now the $\lim _{\epsilon \rightarrow 0}$ of (37), we obtain the system of algebraic-differential equations

38 $$\begin{aligned} \begin{aligned} {\dot{[S]}}&={}- \beta [Sv]+ (1-[S]),\\ {\dot{[SS]}}&={}2 (n[S]-[SS])-2 \beta \frac{n-1}{n}\frac{[SS][Sv]}{[S]},\\ 0&={} \beta [Sv]- \gamma [v],\\ 0&={}-(\gamma +\beta )[Sv]+ \beta \frac{n-1}{n}\frac{[Sv][SS]}{[S]},\\ 0&={}2\beta [Sv]-2 \gamma [vv]. \end{aligned} \end{aligned}$$

The last three equations of (38) are satisfied for $[v]=[Sv]=[vv]=0$. This is exactly the critical manifold of (8), on which the dynamics is described by (32). Using (32), we can show how $\lambda _5$ changes in time, in the slow flow, by deriving its formulation (26) with respect to time, obtaining

$$\begin{aligned} {\dot{\lambda }}_5=\beta \frac{n-1}{n} \frac{2n[S]^2-[SS]([S]+1)}{[S]^2}. \end{aligned}$$

This implies that $\lambda _5$ is increasing if $[SS]<\alpha ([S])$, where the function $\alpha$ is defined by

39 $$\begin{aligned} \alpha (x)=\frac{2nx^2}{x+1}. \end{aligned}$$

**Fig. 5:**
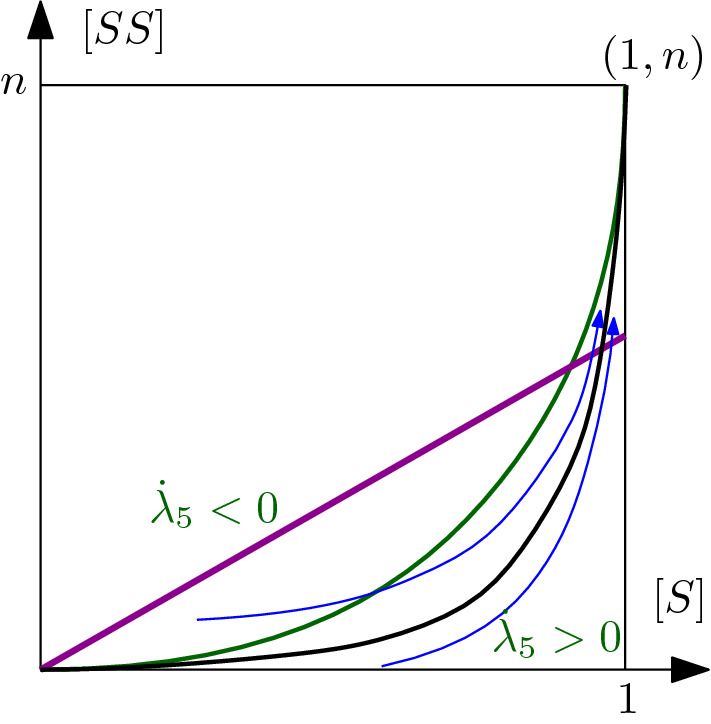
Sign of the derivative in time of $\lambda _5$ under the slow flow. Blue: sketch of orbits starting above/below the green curve $[SS]=\alpha ([S])$. Purple: loss of hyperbolicity line $[SS]=L[S]$ (27). Black: the attracting parabola $\Gamma$ (34). We remark that fast orbits always land below the purple line, which is the region of the rectangle in which the critical manifold is attracting (color figure online)

In Fig. 5 we visualize the behaviour of two orbits in the slow dynamics. We note that, even if an orbit enters the slow flow in a point below the purple line but above the green curve, i.e. in the region where ${\dot{\lambda }}_5<0$, it eventually has to cross the green line before crossing the purple curve, since they represent respectively ${\dot{\lambda }}_5=0$ and $\lambda _5=0$. Hence, any orbit will eventually evolve in the region ${\dot{\lambda }}_5>0$. We prove the following:

#### Proposition 6

The subset $\{ ([S],[SS])\in (0,1)\times (0,n)| {\dot{\lambda }}_5>0 \}$ is forward invariant for system (32).

#### Proof

The normal vector to the curve $\alpha ([S])$ is given by $\nu =(-{\dot{\alpha }}([S]),1)$, with

$$\begin{aligned} {\dot{\alpha }}([S])=\frac{2n[S]([S]+2)}{([S]+1)^2}. \end{aligned}$$

If we take the scalar product of $\nu$ with the vector field *F* given by (32), we obtain

$$\begin{aligned} \nu \cdot F = 2 \left( \frac{n\left( 2[S]^3+3[S]^2-[S]\right) }{([S]+1)^2} -[SS] \right) <2 (\alpha ([S]) -[SS]), \end{aligned}$$

meaning that on the curve $[SS]=\alpha ([S])$, this scalar product is negative, hence orbits approaching the curve from below will not cross it. $\square$

#### Remark 9

By comparing (34) and (39), we notice that the curve $\alpha$ is always above the parabola $\Gamma$; hence, by invariance of $\Gamma$ and Proposition 6, an orbit starting above the parabola will eventually be “squeezed” between $\alpha$ and $\Gamma$.

The following Lemma is also insightful:

#### Lemma 5

Let $\left( [S]^*,[SS]^*\right)$ denote the intersection point between the curve $\{ [SS]=\alpha \left( [S] \right) \}$ and the line $\{ [SS]=L[S] \}$. The endemic equilibrium point converges to $\left( [S]^*,[SS]^*\right)$ as $\epsilon \rightarrow 0$ and $n\rightarrow \infty$.

#### Proof

Let $[S]^*$ denote the solution of $L[S]=\alpha ([S])$. It follows that $[S]^*=\frac{\beta +\gamma }{2\beta n}+{\mathcal {O}}\left( \frac{1}{n^2} \right)$. On the other hand, expanding [*S*] in (31) leads to $[S]=\frac{\beta +\gamma }{\beta n}+{\mathcal {O}}\left( \frac{1}{n^2}\right) +{\mathcal {O}}(\epsilon )$. Therefore $[S]-[S]^*=\frac{\beta +\gamma }{2\beta n}+{\mathcal {O}}\left( \frac{1}{n^2} \right) +{\mathcal {O}}(\epsilon )$, from which the result follows by taking the limits $n\rightarrow \infty$ and $\epsilon \rightarrow 0$. $\square$

The previous Lemma qualitatively tells us that for sufficiently small $\epsilon$ and large enough *n* one expects the endemic equilibrium to be near the intersection point $\left( [S]^*,[SS]^*\right)$. In this case, if the endemic equilibrium is stable, then $\left( [S]^*,[SS]^*\right)$ is a good approximation of such an equilibrium, while if there are limit cycles then these are roughly “centered” at $\left( [S]^*,[SS]^*\right)$. Notice that, in the latter case, limit cycles do not have to be “centered” at $\left( [S]^*,[SS]^*\right)$ if *n* is not large enough. See more details in the forthcoming sections.

### Entry–exit function

Dividing the last three equations of system (36) by $\epsilon$ on both sides, we obtain

40 $$\begin{aligned} \begin{aligned} {[S]}'&={}\epsilon (- \beta [Sv]+ (1-[S]-\epsilon [v])),\\ {[SS]}'&={}\epsilon \left( 2 (n[S]-[SS]-\epsilon [Sv])-2 \beta \frac{n-1}{n}\frac{[SS][Sv]}{[S]}\right) ,\\ {[v]}'&={} \beta [Sv]- \gamma [v],\\ {[Sv]}'&={}-(\gamma +\beta )[Sv]+\epsilon (n[v]-[Sv]-[vv])+ \beta \frac{n-1}{n}[Sv]\left( \frac{[SS]}{[S]}-\epsilon \frac{[Sv]}{[S]}\right) ,\\ {[vv]}'&={}2\beta [Sv]-2 \gamma [vv]+\epsilon \beta \frac{n-1}{n} \frac{[Sv]^2}{[S]}. \end{aligned} \end{aligned}$$

System (40) can be rewritten as

41 $$\begin{aligned} \begin{aligned} x'&={}\epsilon f(x,z)+\epsilon ^2 m(z,w),\\ z'&={}zg(x,z)+\epsilon h(x,z,w),\\ w'&={}-Dw+Az+\epsilon l(x,z), \end{aligned} \end{aligned}$$

where we denote $x:= {{[S]}\atopwithdelims (){[SS]}}$, $z:=[Sv]$, and $w:={{[v]}\atopwithdelims (){[vv]}}$. The critical manifold ${\mathcal {C}}_0=\{z=0, w={{0}\atopwithdelims (){0}}\}$ is invariant for system (41) both when $\epsilon >0$ and $\epsilon =0$. Recall (26); it is clear that $g(x,0)=\lambda _5\lessgtr 0$ when $x\in {\mathcal {C}}_0^A$ or $x\in {\mathcal {C}}_0^R$, respectively.

To control the relation between the starting point of the slow dynamics and the transition point back to the fast dynamics, we are going to employ the entry–exit function (De Maesschalck and Schecter 2016; Liu 2000; Schecter 2008). We cannot apply (Liu 2000, Theorem 2.5), as was done in Jardón-Kojakhmetov et al. (2021), since that requires that the fast dynamics is one-dimensional, while (41) has three fast variables. Instead, we can apply Theorem 4.7 of Liu (2000); see also Schecter (2008, Theorem 2.4), noting that the map $\Pi _0$ was defined at p. 417. We remark, however, that one of the hypotheses necessary for the application of such formula is the separation of the negative eigenvalues from the eigenvalue which causes the loss of stability. To be more precise, let us recall that the non-trivial eigenvalues of the layer equation on ${\mathcal {C}}_0$ are $\lambda _3=-2\gamma$, $\lambda _4=-\gamma$ and $\lambda _5$, see (26). Therefore, the exchange lemma of Theorem 4.7 of Liu (2000) can be applied only to trajectories contained in the portion of ${\mathcal {C}}_0$ in which $\lambda _5>-\gamma$. Recalling (26), we have that $\lambda _5 > -\gamma$ if and only if

42 $$\begin{aligned}{}[SS] > \frac{n}{n-1}[S]. \end{aligned}$$

Thus, let us now assume that a solution of (32) satisfies (42) for all $\tau \ge 0$. Then, denoting with $x_0:=([S]_\infty ,[SS]_\infty )$ and letting $x(\tau ;x_0)$ denote the solution of

43 $$\begin{aligned} {\dot{x}}=f(x,0,0), \qquad x(0)=x_0, \end{aligned}$$

we can implicitly compute the exit time $T_E$ of an orbit on the slow manifold through the integral

44 $$\begin{aligned} \int _{0}^{T_E} g\left( x(\tau ;x_0),0\right) \text {d}\tau =0, \end{aligned}$$

which is simply a rewriting of the entry–exit integral (Liu 2000). Figure 12a, b show two trajectories satisfying (42) for which formula (44) provides a good approximation of the exit time from the slow dynamics.

A natural question concerns conditions that guarantee that (42) holds for all $\tau$. Recalling that for the slow flow we denote the initial conditions by $([S](0),[SS](0))=([S]_\infty ,[SS]_\infty )$, and using (24), we find that (42) holds for $\tau = 0$ if and only if

$$\begin{aligned}{}[S]_\infty > \frac{(n-1)}{n }[S]_0^{\frac{2(n-1)}{n }}[SS]_0^{-1} \approx \frac{(n-1)}{n^2 }[S]_0^{-\frac{2}{n }} \end{aligned}$$

where the last (approximate) equality is obtained as in Remark 8. It is possible that (42) does not hold for some $\tau > 0$ even if holds at $\tau = 0$, but following similar steps as we have just described, one can show that this also happens only if $[S]_\infty$ is sufficiently close to 0. Hence (42) holds for all $\tau \ge 0$ whenever $[S]_\infty$ is large enough. We recall that $[S]_\infty$ can be computed as zero of the function *H*(*x*) given in Proposition 3. Accordingly, let us recall from Proposition 3 that

$$\begin{aligned} H(x)=n\frac{\beta +\gamma }{\beta }\left( x^{\frac{1}{n}}-[S]_0^{\frac{1}{n}}\right) -[SS]_0\left( [S]_0^{\frac{2}{n}-2}x^{1-\frac{1}{n}}-[S]_0^{\frac{1}{n}-1}\right) . \end{aligned}$$

Thus, we see that for $x<[S]_0$, *H* changes proportionally to $\beta$. Then, because *H* increases as $\beta$ also increases, we can use the implicit function theorem to argue that $[S]_\infty$ is a decreasing function of $\beta$, for any fixed $[S]_0\in (0,1)$. The previous fact can also be seen from (21). Hence, from the arguments described above, and recalling that $\beta >\frac{\gamma }{n-2}$ so that $R_1>0$, we conclude that there exists $\beta ^*$ such that (42) is satisfied for all $\tau$ if $\frac{\gamma }{(n-2) }< \beta < \beta ^*$. On the other hand, (42) would not hold if $\beta$ is large enough. In that case, the formula (44) does not provide good approximations for the exit point, see more details in Appendix B. From now on we shall assume that (42) holds.

In order to find $T_E$ from (44), we use (33), introducing, for ease of notation, $A:=[S]_\infty -1 <0$ and $B:=[SS]_\infty -n<0$. Then, (44) becomes

45 $$\begin{aligned} \begin{aligned}&\int _{0}^{T_E}\lambda _5(\tau )\text {d}\tau =\int _{0}^{T_E} \left( -(\gamma +\beta )+ \beta \frac{n-1}{n} \frac{[SS](\tau )}{[S](\tau )} \right) \text {d}\tau =\\&\quad \int _{0}^{T_E} \left( -(\gamma +\beta )+ \beta \frac{n-1}{n}\frac{2A n e^{-2 \tau }(e^{ \tau }-1)+Be^{-2 \tau }+n}{Ae^{- \tau }+1} \right) \text {d}\tau =0, \end{aligned} \nonumber \\ \end{aligned}$$

which gives the following implicit equation for $T_E$

46 $$\begin{aligned} \begin{aligned}&-(\gamma +\beta )T_E+\beta \frac{n-1}{n}\\&\bigg (\frac{Ae^{- T_E}(2An-B)- T_E(B-2A(A+1)n)+(B-A(A+2)n)\ln \left( \frac{A+e^{ T_E}}{A+1}\right) }{A^2 }\\&\quad -\frac{2An-B}{A }\bigg )=0. \end{aligned} \nonumber \\ \end{aligned}$$

Clearly, $T_E=0$ is a solution of (46); the integrand of (45), i.e. $\lambda _5$, along the slow flow, is eventually always increasing, recall Proposition 6.

#### Lemma 6

The exit time $T_E$ is finite for any initial point $([S]_\infty ,[SS]_\infty ) \in {\mathcal {C}}_0^A$.

#### Proof

Recall (45). For small positive values of $\tau$, $\lambda _5(\tau )<0$, since the slow dynamics begins in the attracting region ${\mathcal {C}}_0^A$. Hence, for small values of $\tau \ge 0$ the integral

$$\begin{aligned} \int _{0}^{\tau } \lambda _5(\sigma )\text {d}\sigma <0. \end{aligned}$$

From (46), we observe that

$$\begin{aligned} \lim _{T_E \rightarrow +\infty }\int _{0}^{T_E} \lambda _5(\sigma )\text {d}\sigma =+\infty , \end{aligned}$$

hence there exists at least one finite $T_E$ which satisfies (44). From our previous analysis, we know that $\lambda _5(\tau )=0$ only once during the slow flow, and it remains positive afterwards; hence, such $T_E$ is unique. $\square$

### Application of the entry–exit formula to the parabola

As we have remarked so far, the parabola (34) is of particular interest for the dynamics, even more so for large values of *n*. Hence, we are interested in understanding the entry–exit relation on this specific invariant set. We now consider the evolution, under the slow flow, of the point $([S]_\infty ,[SS]_\infty )=(0,0)$; with these initial conditions, (33) becomes

47 $$\begin{aligned} \begin{aligned} {[S]}(\tau )&={} 1-e^{- \tau },\\ {[SS]}(\tau )&={}n+n e^{-2 \tau } -2ne^{- \tau }=n[S]^2(\tau ). \end{aligned} \end{aligned}$$

Being able to write [*SS*] as a function of [*S*] allows us to compute the exit point for the origin, which in general is not possible, since $\lambda _5$ depends on both slow variables. Combining (47) and (45) we obtain

48 $$\begin{aligned} \begin{aligned}&\int _{0}^{[S]_1}\left( \frac{-(\gamma +\beta )+\beta (n-1)x}{1-x} \right) \text {d}x =0\\&\quad \implies \beta (n-1)\left( 1-[S]_1\right) +(\gamma -(n-2)\beta )\ln \left( 1-[S]_1\right) -\beta (n-1)=0,\\&\quad \implies -\beta (n-1)[S]_1+(\gamma -(n-2)\beta )\ln (1-[S]_1)=0, \end{aligned} \nonumber \\ \end{aligned}$$

where $[S]_1$ indicates the exit point of the orbit which starts at the origin. It can be shown, by direct substitution, that orbits with initial conditions $([S]_\infty ,[SS]_\infty )=([S]_\infty ,n[S]_\infty ^2)$ evolve, under the slow flow (33), along the curve $[SS]=n[S]^2$; moreover, this follows from Lemma 3. The exit point of such an orbit can be computed implicitly, with the same procedure as (48).

#### Lemma 7

Orbits entering the slow flow in a point of the form $([S]_\infty ,[SS]_\infty )=([S]_\infty ,n[S]_\infty ^2)$ exit at a point of the form $([S]_1,n[S]_1^2)$, with $[S]_1$ given by

49 $$\begin{aligned}&-\beta (n-1)[S]_1+(\gamma -(n-2)\beta )\ln (1-[S]_1)\nonumber \\&\quad =-\beta (n-1)[S]_\infty +\left( \gamma -(n-2)\beta \right) \ln \left( 1-[S]_\infty \right) , \end{aligned}$$

which can be equivalently rewritten, introducing for ease of notation $C:=((n-2)\beta -\gamma )/(\beta (n-1))$, as

50 $$\begin{aligned} (1-[S]_1)^C e^{[S]_1} = (1-[S]_\infty )^C e^{[S]_\infty }. \end{aligned}$$

#### Proof

Straightforward computation from the integral in (48), where we substitute the lower bound of integration 0 with a generic $[S]_\infty$. $\square$

#### Lemma 8

If two entry points on the parabola satisfy $[S]_{\infty ,1}<[S]_{\infty ,2}$, then the corresponding exit points satisfy $[S]_{1,1}>[S]_{1,2}$.

#### Proof

**Fig. 6:**
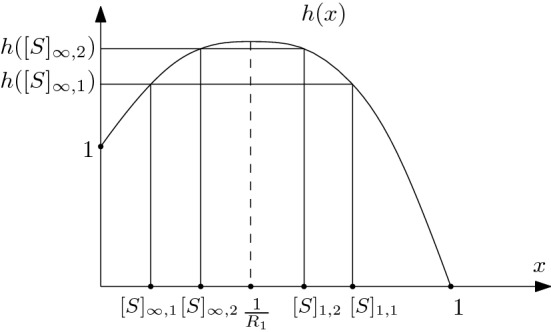
Sketch of the function *h*(*x*) used in the proof of Lemma 8

Recall that the parabola is invariant under the slow flow. The entry–exit relation (50) implicitly defines a function

$$\begin{aligned} h(x):=(1-x)^Ce^x, \end{aligned}$$

meaning that the entry–exit relation can be written as $h([S]_\infty )=h([S]_1)$ (see Fig. 6 for a sketch of the function *h*, and a visualization of the argument of this proof). We observe that $h(0)=1$ and $h(1)=0$. Deriving *h*(*x*), we see that

$$\begin{aligned} h'(x)=(1-x)^{C-1}(1-C-x)e^x>0 \iff x<1-C=\frac{\gamma +\beta }{(n-1)\beta }=\frac{1}{R_1}. \end{aligned}$$

Hence, *h*(*x*) is increasing before $x=1/R_1$, decreasing afterwards. This implies that if $[S]_{\infty ,1}<[S]_{\infty ,2}$ we have that $h([S]_{\infty ,1})<h([S]_{\infty ,2})$, and the corresponding exit points satisfy $[S]_{1,1}>[S]_{1,2}>1/R_1$. $\square$

**Fig. 7:**
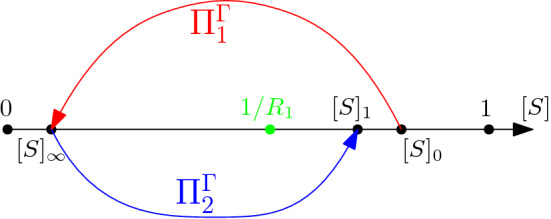
Sketch of the map which relates $[S]_0$ to $[S]_\infty$ (red) and $[S]_\infty$ to $[S]_1$ (blue). The green dot represents the value $1/R_1$: the epidemics can only start for values of $[S]_0>1/R_1$ (color figure online)

The study of the asymptotic behaviour of system (8) is then reduced to two 2-dimensional maps, from ${\mathcal {C}}_0$ to itself; specifically, we define $\Pi _1([S]_0,[SS]_0)=([S]_\infty ,[SS]_\infty )$ and $\Pi _2([S]_\infty ,[SS]_\infty )=([S]_1,[SS]_1)$. We now explain the rationale behind introducing the one-dimensional maps $\Pi _1^\Gamma$ and $\Pi _2^\Gamma$ obtained by restricting the domain and approximating the range of $\Pi _1$ and $\Pi _2$ to the parabola $\Gamma$ (see Fig. 7). Indeed, Remark 8 and Lemma 3 show that the parabola $\Gamma$ is close to being invariant for the map $\Pi _2 \circ \Pi _1$ and it is well known that the occurrence of near one-dimensional return maps is an important theme in multiple time scale systems (Bold et al. 2003; Guckenheimer et al. 2006; Kuehn 2011; Medvedev 2005).

Next, consider a point with [*S*] coordinate $[S]_0$, ${\mathcal {O}}(\epsilon )$ away from the parabola $\Gamma$ (34), in the repelling part of the critical manifold. Its image $[S]_\infty$ under the fast flow, which defines the map $\Pi _1$ sketched in Fig. 7, is given by (35). We notice that this value depends on both $\beta$ and $\gamma$, as well as on *n*. For *n* large enough, the entry point in the slow flow will be close to the parabola, as argued in Remark 8; hence, we will be able to compute its exit point $[S]_1$ using (49), which again depends explicitly on all the parameters of the system in a highly non-trivial way. This is different from the SIRWS model studied in Jardón-Kojakhmetov et al. (2021), in which there was a clear separation between fast parameters, which dictated the fast dynamics, and had no influence on the slow one, and slow parameters, which characterised the viceversa. The map $\Pi _2$ in Fig. 7 sketches the relation between the entry point $[S]_\infty$ and its corresponding exit point $[S]_1$, i.e. (50).

Depending on the relative position of $[S]_0$ and $[S]_1$, we might be able to deduce the asymptotic behaviour of the system. However, the high dimensionality of the layer equation and the complex implicit relation between $[S]_0$ and $[S]_\infty$ (recall (35)) hinders the analysis of the system with non-numerical tools.

We proceed now to a bifurcation analysis of system (8), and finally, with a technique similar to the one detailed Jardón-Kojakhmetov et al. (2021, Sec. 3.4.1), to numerically investigate the existence of periodic orbits by concatenation of fast and slow pieces. We stress the versatility of the numerical argument we present, which is similar to the one we used in Jardón-Kojakhmetov et al. (2021), applied now to a higher dimensional system.

## Bifurcation analysis and numerical simulations

In this section, we carry out a bifurcation analysis for the behaviour of system (8), which will then be verified by numerical simulations and by a geometrical argument. Bifurcation analysis is done on system (8), which for small values of $\epsilon$ is stiff (as we showed in Proposition 5, the slow manifold is exponentially close to the critical manifold), while the numerical simulation concerns a combination of systems (14) and (32), which are both non-stiff.

It is important to notice that, even though the layer system (14) converges to the critical manifold forwards in time, the slow flow (32) would converge to the point $([S],[SS])=(1,n)$ if we let it evolve freely; the derivation of the exit time (46) is fundamental, in this setting, to carry out a meaningful numerical exploration of the model.

Without loss of generality, we set $\gamma$, which is the inverse of the average infection interval, to 1; this simply amounts to an ${\mathcal {O}}(1)$ rescaling of time, and we rescale the other parameters accordingly, keeping however the same symbols, for ease of notation. System (8) then has only three parameters, namely $\epsilon$, *n* and $\beta$.

**Fig. 8:**
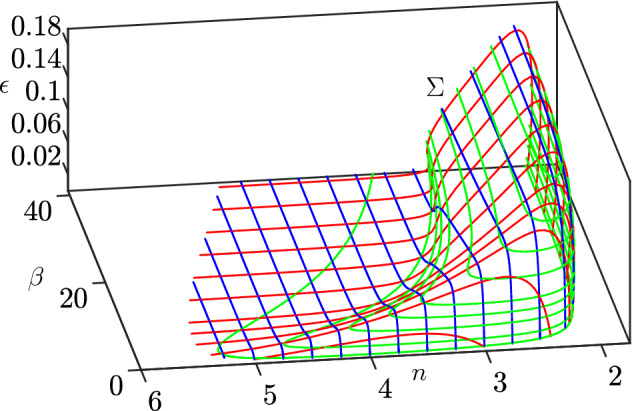
A skeleton of the bifurcation surface $\Sigma$. Green (respectively, red and blue) curves correspond to constant values of $\epsilon$ (respectively, $\beta$ and *n*). We notice that, for values of $n\ge 6$, system (8) converges to the endemic equilibrium (31) regardless of the value of $\epsilon$ and $\beta$ (color figure online)

**Fig. 9:**
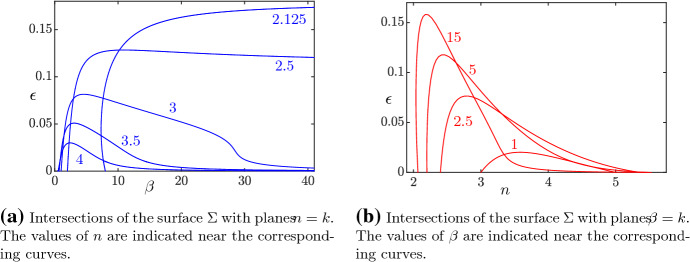
A subset of the blue and red curves from Fig. 8 (color figure online)

**Fig. 10:**
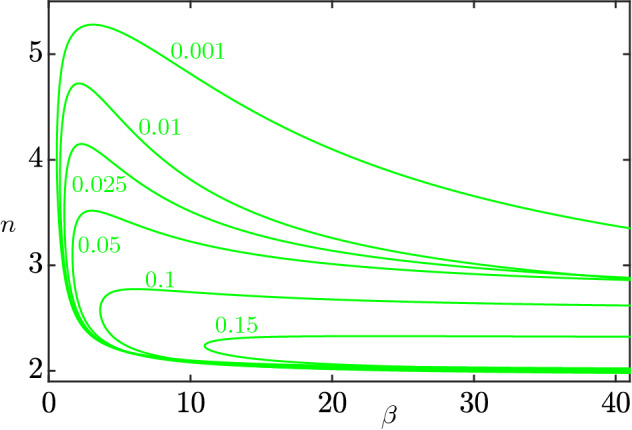
Intersections of the surface $\Sigma$ with planes $\epsilon =k$. The values of $\epsilon$ are indicated near the corresponding curves

Using MatCont (Dhooge et al. (2008)), we are able to completely characterize system (8) through numerical bifurcation analysis. We only consider the first octant of ${\mathbb {R}}^3$, for the biological interpretation of the parameters. Numerical analysis shows the existence of a Hopf surface $\Sigma$, whose “skeleton” is depicted in Fig. 8. For values of the parameters between the plane $\epsilon =0$ and $\Sigma$, the system exhibits a stable limit cycle, while for values above $\Sigma$, the system exhibits convergence to the endemic equilibrium (31). Our bifurcation analysis suggests the existence of a value $\epsilon ^* \approx 0.18$ such that, for $\epsilon >\epsilon ^*$, the system only exhibits convergence to the endemic equilibrium, regardless of the values of $\beta$ and *n*. To make Fig. 8 more readable, we provide intersections of the surface $\Sigma$ with some planes $n=k$ (Fig. 9a), $\beta =k$ (Fig. 9b), and finally $\epsilon = k$ (Fig. 10).

As in Jardón-Kojakhmetov et al. (2021), we see an expansion of the parameter region which exhibits stable limit cycles as $\epsilon$ decreases, see Fig. 10. This means that, as $\epsilon$ decreases, i.e. as the ratio between the average lengths of the infectious phase and the immunity interval decreases, we are more likely to observe occurrence of stable limit cycles in the disease dynamics. We do not observe, however, a divergence in the *n* direction, as the limit as $\epsilon \rightarrow 0$ of the surface contained in the green curves of Fig. 10 is still bounded.

In order to verify the accuracy of the surface $\Sigma$, we investigate the system via a numerical implementation of the same geometrical argument used in Jardón-Kojakhmetov et al. (2021, Sec. 3.4.1). There, we numerically showed the existence of a candidate orbit by concatenating heteroclinic orbits of the layer equation, from the critical manifold to itself, and orbits of the slow flow, truncating each at the corresponding exit time. The system studied in Jardón-Kojakhmetov et al. (2021) was 3-dimensional, but the slow flow evolved on a 2-dimensional plane in ${\mathbb {R}}^3$; as we showed thus far, system (8) is characterized by a 2-dimensional slow manifold, as well. We now briefly recall the construction of the geometrical argument.

For this description, let us fix $\epsilon = 0$, $n=3$, and consider different values of $\beta$. A candidate starting point $[{{\hat{S}}}]$ for a periodic orbit was found by iterating multiple times the layer system (14) and the slow flow (32), which is integrated only for time equal to the exit time (46). These are the maps $\Pi _1$ and $\Pi _2$, respectively, in Sect. 3.7, so in other words we have $([{{\hat{S}}}], [{\hat{SS}}] )= \Pi ^m(([S]_0,[SS]_0))$ where $([S]_0,[SS]_0)$ is an arbitrary initial point, $\Pi = \Pi _2 \circ \Pi _1$ and *m* is large enough that further iterations do not yield substantial changes.

**Fig. 11:**
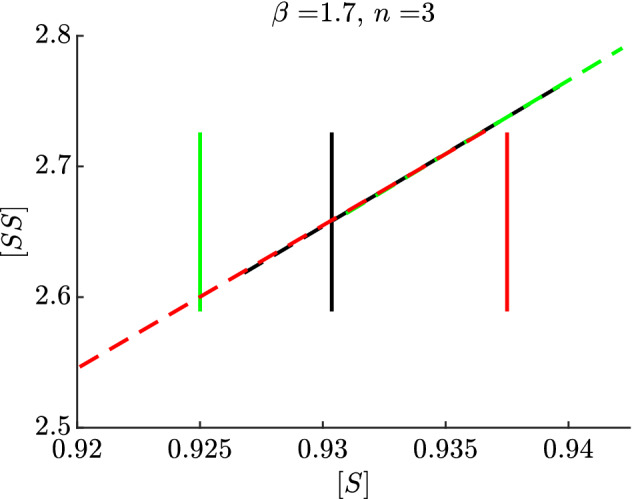
Representation of three $J_1$s (solid lines) and the respective $J_3$s (dashed) after a concatenation of a fast loop and a slow flow stopped at the exit time (46). The intersection of the black lines represent the approximate exit point of the candidate limit cycle. For $J_1$ corresponding to a smaller $[S]_0$ (green), the exit segment yields greater values of $[S]_1$. Viceversa, for $J_1$ corresponding to a larger $[S]_0$ (red), the exit segment yields smaller values of $[S]_1$. The interpretation is that only in the case of transversal intersection (black lines) we have a candidate singular limit cycle (color figure online)

Once we have obtained the candidate value $[{{\hat{S}}}]$, we define a small interval $J_1$ in the [*SS*] coordinate around $[{\hat{SS}}]$. Next, the interval $J_2$ is obtained as the evolution of $J_1$ under the layer equation, that is $J_2=\Pi _1(J_1)$. Analogously, we then obtain the interval $J_3$ as the evolution of $J_2$ under the slow flow *for a time precisely given by* $T_E$, that is $J_3 =\Pi _2(J_2)$. In Jardón-Kojakhmetov et al. (2021) we have shown that if $J_3$ intersects $J_1$ transversally, then the perturbed system, for $\epsilon >0$ small enough, exhibits a limit cycle. Furthermore, if the map $\Pi =\Pi _2\circ \Pi _1$ is a contraction, then the limit cycle is locally stable. Here we provide an additional numerical illustration of our argument, see Fig. 11. The black solid and dashed lines depict, respectively, a choice of $J_1$ and the corresponding $J_3$. We see that these two intervals intersect transversally. This hints to the possibility of a singular cycle with exit point close to the intersection point. To be more specific, when taking an interval $J_1$ corresponding to $[S]_0$ smaller than $[{{\hat{S}}}]$ (green solid line), the interval $J_3$ we obtain (green dashed line) includes values of [*S*] larger than $[S]_0$. Conversely, when $J_1$ includes values $[S]_0$ larger than $[{{\hat{S}}}]$ (red solid line), the interval $J_3$ we obtain (red dashed line) includes values of [*S*] smaller than $[S]_0$. In either of these two latter cases, the intervals do not intersect. This behaviour provides numerical evidence that there exists a point $p=([S],[SS])$ close to $([{{\hat{S}}}], [{\hat{SS}}] )$ such that $\Pi (p) = p$. That is, *p* is a fixed point of the map $\Pi$. In this way, the concatenation of an orbit of the layer system that is backward asymptotic to *p* and an orbit of the slow subsystem with exit point at *p* is what we call *a singular cycle*. Regarding stability, since we have shown that the slow flow is a contraction, and that the flow of the layer system is not expanding, we can conclude that the singular cycle is attracting.

**Fig. 12:**
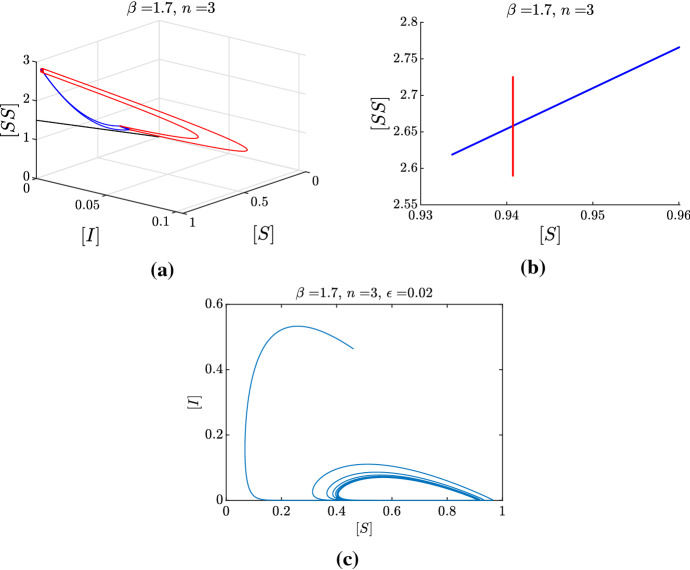
Numerical illustration of the geometrical argument used to show existence of a limit cycle. **a** Evolution under the layer equation (red) of a small interval $J_1$. The corresponding image defines the entry interval $J_2$ on the critical manifold. The evolution of each point of $J_2$ under the slow flow, and stopped at its exit time, is shown in blue and defines the exit interval $J_3$. Notice that the blue curves lie on the [*S*], [*SS*] plane, while the red curves represent a fast excursion in the region $[I],[SI],[II]>0$. The black line represents the border of (44). **b** Zoom on the positions of $J_1$ (red) and $J_3$ (blue) on the critical manifold. **c** Projection on the ([*S*], [*I*]) plane of a numerical simulation of system (8) from a random initial condition, exhibiting convergence to a stable limit cycle predicted by our geometrical argument (color figure online)

Figure 12a, b depict the numerical realization of the two limit systems for a choice of $(n,\beta )$ for which we do expect limit cycles: indeed, $J_1$ and $J_3$ intersect transversely, and bifurcation analysis confirms that, for this choice of the parameters, there is a stable limit cycle for $\epsilon >0$ sufficiently small. Figure 12c, is the projection on the ([*S*], [*I*])-plane of an orbit of system (8), starting from a random initial condition. As we expected, for $\epsilon$ sufficiently small, the perturbed system exhibits a stable limit cycle, as argued from the limiting situation depicted in Fig. 12 a, b.

**Fig. 13:**
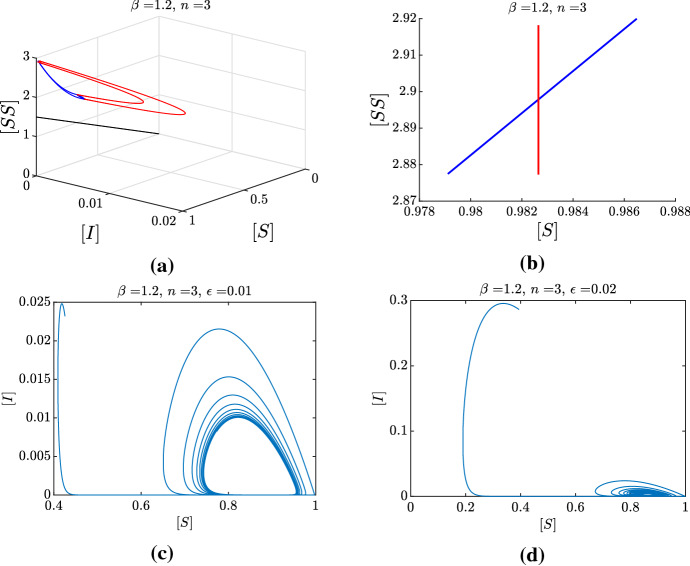
Numerical illustration of the geometrical argument, with a particular focus on the effect of increasing $\epsilon$ on the dynamics. **a, b** Correspond to the singular flows as in Fig. 12, here for the choice of parameters $\beta =1.2$ and $n=3$. **c, d** The projections on the ([*S*], [*I*]) plane of two numerical simulations of system (8) starting from a random initial condition, for $\epsilon =0.01$ and $\epsilon =0.02$, respectively. Notice that since the intervals $J_1$ and $J_3$ intersect transversely, we expect to find a stable limit cycle for $\epsilon >0$ sufficiently small. This is indeed true for $\epsilon =0.01$, but no longer true for $\epsilon =0.02$. In other words, for this choice of parameters, $\epsilon =0.02$ is not sufficiently small so that our geometric argument holds

Moreover, we remark on the necessity of $\epsilon$ being “small enough”, with this condition being represented by the surface $\Sigma$, for the above argument to hold. In particular, in Fig. 13, we show how increasing $\epsilon$ from 0.01 to 0.02 destroys the limit cycle at corresponds, instead, to convergence to the endemic equilibrium. Hence, for this specific realization, $\epsilon =0.01$ is small enough, whereas $\epsilon =0.02$ is already too large. The threshold for $\epsilon$ is given by the intersection of the surface $\Sigma$ and the line $\{ n=3, \beta =1.2 \}$ in the $(n,\beta ,\epsilon )$-space represented in Fig. 8.

## Discussion and outlook

We have analysed the behaviour of an SIRS model for epidemics on networks, exploiting Geometric Singular Perturbation Theory (GSPT), similarly to what performed in Jardón-Kojakhmetov et al. (2021), in a way appropriate to a system in a nonstandard singularly perturbed form.

From the point of view of applications, the main result found, through bifurcation analysis and geometric numerical arguments, is that, for a significant open subset of the parameter space, the network model exhibits stable limit cycles. This result is in sharp contrast to the global convergence to equilibrium of solutions to the standard (random mixing) SIRS model (Hethcote 1976; O’Regan et al. 2010; Jardón-Kojakhmetov et al. 2021).

Stable periodic cycles are found the value of *n*, the number of neighbours every individual has, is between 3 and 5 included. It is not surprising that a small value of *n* is needed, since for large *n* the model approximates a random mixing model, which, as stated above, exhibits global convergence to equilibrium. On the other hand, $n \le 5$ may appear a strongly restrictive condition for actual contact networks; however, it must be considered that in the model all contacts are assumed to have the same strength, while in reality individuals may have a limited number of strict contacts (say, three to five very close friends) plus a number of infrequent contacts. An interesting question would be whether the same result would hold with such a network model with weighted connections. Thus, it is clear that there is a strong motivation to use techniques from GSPT to investigation more complex network models, since the current analysis unveiled asymptotic behaviours which are impossible in the corresponding system under the random mixing assumption.

The results shown here were obtained for a deterministic system representing the pair approximation (Kiss et al. 2017) of a stochastic network model. A natural question is whether the periodic solutions, identified in the pair-approximation model, correspond to a recognizable pattern in the network simulations as well. Preliminary numerical explorations seem to suggest that indeed network simulations fluctuate around a closed orbit when the deterministic model predicts an attracting periodic solution. We plan to pursue further these investigations, that are however hampered, for $\epsilon$ particularly small, by the necessity of simulating a very large network to avoid the infection dying out in the long time orbits spend near $[I]=0$.

This last observation raises a general issue concerning the applicability of the model. Our analysis (Lemma 6) shows that the slow flow takes a finite time (the time passing between two consecutive outbreaks of the infection) in the slow scale, hence of the order ${\mathcal {O}}(1/\epsilon )$ in the fast time scale. During that period the value of *I* (representing the fraction of infected nodes) is exponentially (in $\epsilon$) close to 0; hence, in a more realistic stochastic model, complete extinction of the infection would be likely, unless population size *N* is very large or there exists the possibility of external reintroduction of the infection. In the context of stochastic models, it would probably be interesting considering joint limits in which $\epsilon \rightarrow 0$ while $N \rightarrow \infty$, as in the discussion of critical community size in Diekmann et al. (2013).

We stress the versatility of our geometric procedure, which gives us a numerical intuition of the asymptotic behaviour of a stiff system, i.e. system (8) with $0<\epsilon \ll 1$, without having to *actually* integrate it, but through simple integration of the corresponding two non-stiff limit systems, which we derived through the use of GSPT. This is particularly important for the high(er) dimensionality of the system, which hinders analytical results on the perturbed system. In particular, the same strategy is likely to generalize to more complicated network-based ODE models derived from moment closure.

We must acknowledge that one of the main tools used in the analysis, the entry–exit map, requires an extra assumption (regarding the order of the negative eigenvalues of the critical manifold) to ensure that the fast dynamics near the critical manifold can be approximately one-dimensional. The assumption holds if the contact rate $\beta$ is not too large, but fails for large values of $\beta$. When this happens (see Appendix B), the formula (44) to predict the exit point is no longer correct, but we see numerically that periodic solutions still exist in that case too. This stresses the need to develop methods to be able to deal with the case when the fast dynamics is not one-dimensional.

As mentioned above, we expect that for $n\rightarrow +\infty$ the system approaches the simple epidemic model with random mizing; however, this has not been rigorously established. One intermediate step between having two independent perturbation parameters (i.e., $\epsilon \rightarrow 0$ and $n \rightarrow +\infty$) could be to couple *n* and $\epsilon$, for example taking $n={\mathcal {O}}(1/\epsilon ^\alpha )$, for some $\alpha >0$. However, this goes beyond the scope of this project, and we leave this as a prompt for future research.

## Figures and Tables

**Fig. 14:**
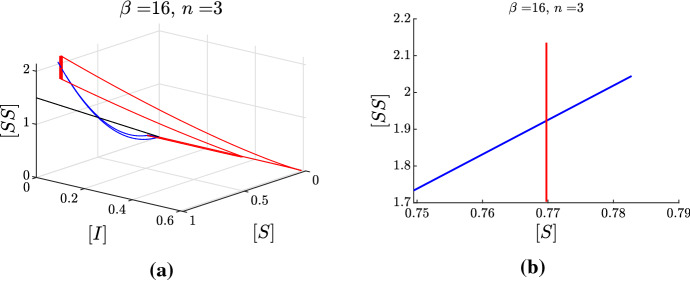
**a** Evolution under the layer system (red) of a small interval $J_1$. Its image defines the entry interval $J_2$ on the critical manifold. The evolution of each point of $J_2$ under the slow flow, stopped at the exit time provided by formula (46), is shown in blue. The evolution of $J_2$ defines the exit interval $J_3$. The black line represents the border of (42): notice that the blue orbits partially lie below that line. **b** Zoom on the relative position of $J_1$ and $J_3$ on the critical manifold. Since these intervals intersect transversely, our geometrical argument predicts a limit cycle with exit point ${\mathcal {O}}(\epsilon )$ close to $[S]\approx 0.77$

**Fig. 15:**
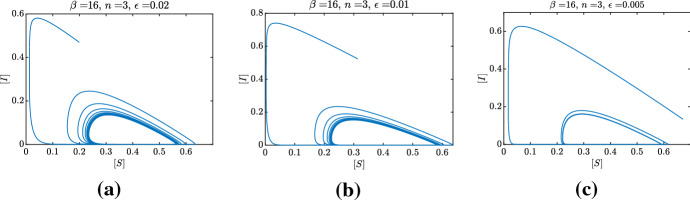
Simulations of system (8) for $n=3$, $\beta = 16$, random initial conditions, and decreasing values of $\epsilon$. In this case the exit point for the limit cycle is $[S]\approx 0.58$, which is not close to the one predicted by our entry–exit formulation, compare with Fig. 14

## Appendix A

Recall Proposition 5. In this section, we explicitly show that the slow manifold of system (8) is exponentially close to the critical manifold ${\mathcal {C}}_0$ (25).

### Proof

First of all, we notice that $[I]={\mathcal {O}}(\epsilon )$ implies $[SI],[II],[IR]={\mathcal {O}}(\epsilon )$; recall (7b). Proceeding as in Taghvafard et al. (2019), we propose the expansion

51a $$\begin{aligned}{}[I]&={}f_1([S],[SS])\epsilon +{\mathcal {O}}(\epsilon ^2), \end{aligned}$$

51b $$\begin{aligned}&={}f_2([S],[SS])\epsilon +{\mathcal {O}}(\epsilon ^2), \end{aligned}$$

51c $$\begin{aligned}&={}f_3([S],[SS])\epsilon +{\mathcal {O}}(\epsilon ^2), \end{aligned}$$

where the functions $f_i$ are as smooth as necessary. We use these expansions in the respective equations for $[I]', [SI]', [II]'$ in system (8), and match the corresponding powers of $\epsilon$.

Here we show the details with [*I*]. For ease of notation, we omit arguments of the functions $f_i$ everywhere. We need to solve

$$\begin{aligned}{}[I]' = \left( \frac{\partial f_1}{\partial [S]} [S]' + \frac{\partial f_1}{\partial [SS]}[SS]' \right) \epsilon + {\mathcal {O}}\left( \epsilon ^2\right) = \beta [SI] - \gamma [I], \end{aligned}$$

which becomes

52 $$\begin{aligned} \begin{aligned}&\left( \frac{\partial f_1}{\partial [S]}\left( -\beta f_2 \epsilon + \epsilon \left( 1-[S]-f_1 \epsilon \right) + {\mathcal {O}}\left( \epsilon ^2\right) \right) + \frac{\partial f_1}{\partial [SS]}\left( 2\epsilon \left( n[S]-[SS]-f_2 \epsilon \right) \right. \right. \\&\quad \left. \left. -2\beta \frac{n-1}{n}\frac{[SS]f_2 \epsilon }{[S]}\right) \right) \epsilon +{\mathcal {O}}(\epsilon ^2) = \beta f_2 \epsilon -\gamma f_1 \epsilon + {\mathcal {O}}\left( \epsilon ^2\right) . \end{aligned} \end{aligned}$$

The LHS of (52) is ${\mathcal {O}}(\epsilon ^2)$, while RHS of (52) is ${\mathcal {O}}(\epsilon )$; this means that, at first order in $\epsilon$, we have to solve RHS = 0 at first order in $\epsilon$, i.e. ignoring the contribution which is ${\mathcal {O}}(\epsilon ^2)$.

From the equation for $[I]'$ we see that

$$\begin{aligned} 0=\beta f_2 \epsilon - \gamma f_1 \epsilon \implies f_1 = \frac{\beta }{\gamma } f_2. \end{aligned}$$

The same arguments can be applied for [*SI*] and [*II*]. From the equation for $[SI]'$ we have

$$\begin{aligned} 0= & {} -(\gamma +\beta ) f_2 \epsilon + \epsilon ^2 \left( nf_1-f_2-f_3\right) \\&+\frac{n-1}{n} \beta f_2 \epsilon \left( \frac{[SS]}{[S]} - \frac{f_2 \epsilon }{[S]}\right) \implies f_2= 0 \implies f_1= 0. \end{aligned}$$

From the equation for $[II]'$

$$\begin{aligned} 0=2\beta \epsilon f_2-2\gamma \epsilon f_3+2\epsilon ^2\beta \frac{n-1}{n}\frac{f_2^2}{[S]} \implies f_3= 0. \end{aligned}$$

This shows that, at first order in $\epsilon$, $f_1 = f_2 = f_3 = 0$. So, in the first order in $\epsilon$, the slow manifold is still $[I]=[SI]=[II]=0$.

We now prove by induction that, for any $k \in {\mathbb {N}}$, the slow manifold is exactly 0 in the expansion up to $\epsilon ^k$. By assumption, we can write $[I]=g_1([S],[SS],[SR])\epsilon ^k+{\mathcal {O}}(\epsilon ^{k+1})$, $[SI]=g_2([S],[SS],[SR])\epsilon ^k+{\mathcal {O}}(\epsilon ^{k+1})$, $[II]=g_3([S],[SS],[SR])\epsilon ^{k}+{\mathcal {O}}(\epsilon ^{k+1})$.

Proceeding as above, all the LHSs will be ${\mathcal {O}}(\epsilon ^{k+1})$, while the RHSs will be ${\mathcal {O}}(\epsilon ^k)$, meaning we still have to solve RHS = 0.

From the equation for $[I]'$ (omitting, once again, all the arguments of $g_i$ everywhere, for ease of notation):

$$\begin{aligned} 0=\beta g_2 \epsilon ^k - \gamma g_1 \epsilon ^k \implies g_1 = \frac{\beta }{\gamma } g_2. \end{aligned}$$

From the equation for $[SI]'$:

$$\begin{aligned} \begin{aligned} 0&=-(\gamma +\beta ) g_2 \epsilon ^k + \epsilon ^{k+1} \left( ng_1-g_2-g_3\right) +\frac{n-1}{n} \beta g_2 \epsilon ^k \left( \frac{[SS]}{[S]} - \frac{g_2 \epsilon ^k}{[S]}\right) \\&\quad \implies g_2= 0 \implies g_1= 0. \end{aligned} \end{aligned}$$

From the equation for $[II]'$:

$$\begin{aligned} 0=2\beta g_2 \epsilon ^{k} - 2\gamma g_3 \epsilon ^{k} +\beta \frac{n-1}{n} \epsilon ^{2k}\frac{g_2^2 }{[S]} \implies g_3 = 0. \end{aligned}$$

This shows that the slow manifold is exponentially close in $\epsilon$ to the critical manifold $[I]=[SI]=[II]=0$. $\square$

## Appendix B

Let us recall that our use of the entry–exit formula and our geometric argument to find candidates for limit cycles require the assumption that $[SS]>\frac{n}{n-1}[S]$, as already given in (42). This is because, in such a case, the non-trivial eigenvalues of the critical manifold are arranged as $-2\gamma<-\gamma <\lambda _5$. In this situation we can simply disregard the contribution of the more negative eigenvalues. The situation is quite different if the above ordering does not hold along orbits of the slow subsystem. In this Appendix, we provide numerical evidence that the formulas derived in Sect. 3.6 fail to reliably predict the exit point whenever the above assumption does not hold.

We start by considering a pair of parameters ($\beta =16$, $n=3$) for which our bifurcation analysis predicts that orbits converge to a stable limit cycle. In Fig. 14, we depict the same procedure as in in Fig. 12, and we indicate with a black line the set $\left\{ [SS]=\frac{n}{n-1}[S] \right\}$. Notice that $J_2$, and part of the orbits corresponding to the slow flow, lie in the region defined by $[SS]<\frac{n}{n-1}[S]$. That is, for these orbits the important above assumption does not hold. Therefore, the exit time presented in Sect. 3.6 may not be correct, as we indeed show below.

We now compare the theoretical result we would obtain by applying the entry–exit function to numerical simulations of the perturbed system (8), shown in Fig. 15. The simulation confirms the presence of a stable limit cycle, but the exit point (the largest [*S*] value in the cycle) is not ${\mathcal {O}}(\epsilon )$ close to the one predicted by the entry–exit formula ($[S]\approx 0.77$). These numerical simulations suggest that the straightforward application of the entry–exit formula to orbits crossing the line $\left\{ [SS]=\frac{n}{n-1}[S] \right\}$ provides wrong numerical estimates of the exit point. This is likely due to the fact that the ordering of the eigenvalues is not preserved along the orbits. Thus, we conjecture that the interplay between the two attracting but constant fast directions and $\lambda _5$ has a non-trivial role in the entry–exit map.

A first step towards resolving the issue described in this Appendix would be to understand how the ordering of the multiple non-trivial eigenvalues of the critical manifold influences the dynamics and especially what the corresponding entry–exit function would be.
